# Suberitamides A–C, Aryl Alkaloids from a *Pseudosuberites* sp. Marine Sponge that Inhibit Cbl-b Ubiquitin Ligase Activity

**DOI:** 10.3390/md18110536

**Published:** 2020-10-28

**Authors:** Chang-Kwon Kim, Dongdong Wang, Brice A. P. Wilson, Josep Saurí, Donna Voeller, Stanley Lipkowitz, Barry R. O’Keefe, Kirk R. Gustafson

**Affiliations:** 1 Molecular Targets Program, Center for Cancer Research, National Cancer Institute, Frederick, MD 21702-1201, USA; chang-kwon.kim@nih.gov (C.-K.K.); dongdong.wang@nih.gov (D.W.); brice.wilson@nih.gov (B.A.P.W.); okeefeba@mail.nih.gov (B.R.O.); 2 Structure Elucidation Group, Analytical Research and Development, Merck & Co., Inc., Boston, MS 02115, USA; josep.sauri.jimenez@merck.com; 3 Women’s Malignancies Branch, Center for Cancer Research, National Cancer Institute, Bethesda, MD 20892-1578, USA; Donna.Voeller@nih.gov (D.V.); lipkowis@mail.nih.gov (S.L.); 4 Natural Products Branch, Developmental Therapeutics Program, Division of Cancer Treatment and Diagnosis, National Cancer Institute, Frederick, MD 21701-1201, USA

**Keywords:** marine sponge, *Pseudosuberites* sp., suberitamides, PIP HSQMBC IPAP, Cbl-b ubiquitin ligase inhibition

## Abstract

Three new aryl alkaloids named suberitamides A–C (**1**–**3**), were isolated from an extract of the marine sponge *Pseudosuberites* sp. collected along the coast of North Carolina. Their planar structures were established by extensive nuclear magnetic resonance (NMR) and mass spectrometry (MS) analysis. To assign the challenging relative configuration of the saturated five-membered ring in suberitamide A (**1**), a simple and efficient NMR protocol was applied that is based on the analysis of 2- and 3-bond ^1^H-^13^C spin-spin coupling constants using a PIP (pure in-phase) HSQMBC (heteronuclear single quantum multiple bond correlation) IPAP (in-phase and anti-phase) experiment. Suberitamides A (**1**) and B (**2**) inhibited Cbl-b, an E3 ubiquitin ligase that is an important modulator of immune cell function, with IC_50_ values of approximately 11 μM.

## 1. Introduction

The Casitas B-lineage lymphoma proto-oncogene b (Cbl-b) is a RING finger E3 ubiquitin ligase that has been identified as a negative regulator of T-cells, NK cells, B cells, and different types of myeloid cells [1,2,3,4]. It also regulates innate immune responses and plays an important role in host defense toward pathogens [5]. Since Cbl-b suppresses activation of diverse immunologic responses, it may represent a potential therapeutic target for the management of human immune-related disorders such as autoimmune diseases and allergic inflammation, as well as impacting the immune response to infections and tumors [6,7]. Compounds that can inhibit the ubiquitin ligase activity of Cbl-b may provide lead structures for the development of immune-modulating therapeutic interventions.

**Figure 1:**
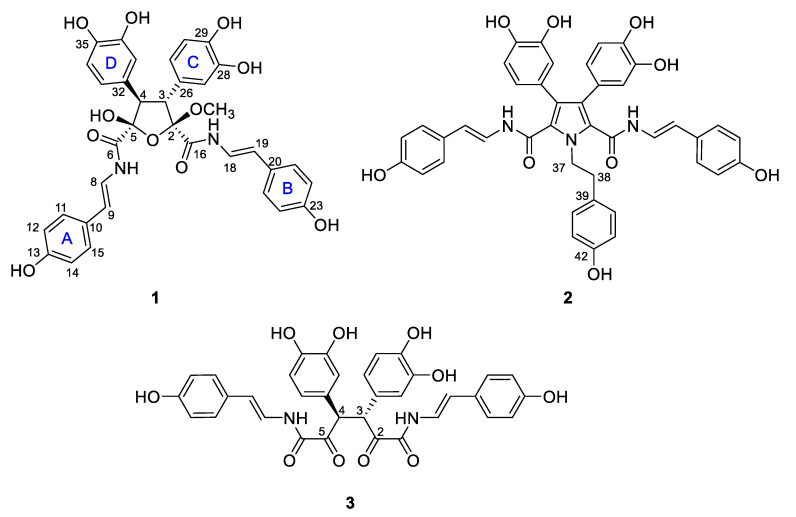
Structures of suberitamides A–C (**1**–**3**).

In conjunction with ongoing NCI (National Cancer Institute) anticancer natural product discovery efforts [8,9], the extract of a North Carolina collection of the marine sponge *Pseudosuberites* sp. was screened, and showed significant activity in an assay for inhibitors of Cbl-b ubiquitin ligase [10]. Bioassay-guided fraction of the extract provided three new aryl alkaloids that were named suberitamides A–C (**1**–**3**) (Figure 1). The lead compound, suberitamide A (**1**) possesses a saturated five-membered ring which exists as numerous puckered conformations due to the inherent conformational flexibility of these rings. Assigning the relative configurations of contiguous stereogenic centers in these systems can be problematic, but for **1** they were established by a simple and effective *J*-based methodology using the PIP (pure in-phase) HSQMBC IPAP experiment [11]. This NMR technique allows the accurate determination of heteronuclear coupling constants (^n^*J*_CH_, n > 1) in a broadband manner while providing easy-to-analyze, pure in-phase lineshapes, and it is particularly useful for measuring couplings between protons and non-protonated carbons. We successfully applied this methodology to determine the relative configuration for the saturated five-membered ring in suberitamide A (**1**). Suberitamides B (**2**) and C (**3**) were readily identified as symmetric molecules from their NMR and ESI–MS data. Detailed 2D NMR analysis revealed suberitamide B (**2**) as a more highly substituted analogue of **1** with a pyrrole moiety in the central position of the molecule, while suberitamide C (**3**) was an oxidized, ring-opened homologue of **1**. Compounds **1** and **2** inhibited Cbl-b enzymatic activity in an in-vitro ubiquitin ligase assay.

## 2. Results and Discussion.

**Figure 2:**
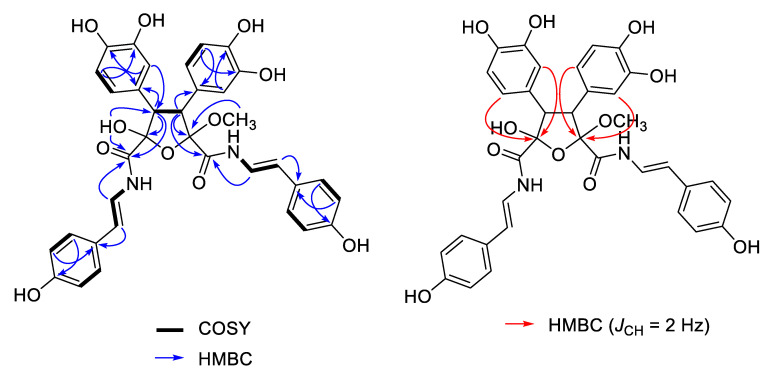
Selected 2D NMR correlations for suberitamide A (**1**). HMBC (heteronuclear multiple bond correlation) experiments were optimized for *J*_CH_ = 8.0 Hz (blue arrows, measured in DMSO-*d*_6_) or *J*_CH_ = 2.0 Hz (red arrows, measured in CD_3_OD).

The molecular formula of suberitamide A (**1**) was determined by HRESIMS to be C_35_H_32_N_2_O_11,_ with 21 degrees of unsaturation. Detailed examination of the ^1^H and ^13^C NMR data (Table 1) aided by 2-D NMR experiments, revealed that this compound possessed two *para*-hydroxystyrylamide (*p*HSA) moieties (A and B rings). The ^1^H NMR spectrum (DMSO-*d*_6_) indicated the presence of two *trans* disubstituted double bonds with signals at *δ*_H_ 7.29 (1H, dd, *J* = 14.6, 10.2 Hz) and 6.43 (1H, d, *J* = 14.6 Hz), along with 7.04 (1H, dd, *J* = 14.6, 10.2 Hz) and 6.29 (1H, d, *J* = 14.6 Hz). Incorporation of these olefins into two enamide functionalities was apparent from COSY correlations between H-8/7-NH (*δ*_H_ 10.52) and H-18/17-NH (*δ*_H_ 10.50), while two non-equivalent *p*-hydroxyphenyl groups were also established with *δ*_H_ 7.24 (2H, d, *J* = 8.4 Hz) coupled to 6.72 (2H, d, *J* = 8.4 Hz) and 7.18 (2H, d, *J* = 8.4 Hz) coupled to 6.70 (2H, d, *J* = 8.4 Hz). Furthermore, two typical ABM spin systems at *δ*_H_ 6.55 (1H, d, *J* = 1.8 Hz), 6.51 (1H, d, *J* = 8.4 Hz), and 6.38 (1H, dd, *J* = 8.4, 1.8 Hz), along with 6.52 (1H, d, *J* = 1.8 Hz), 6.50 (1H, d, *J* = 8.4 Hz) and 6.36 (1H, dd, *J* = 8.4, 1.8 Hz) were indicative of two dihydroxyphenyl groups (rings C and D) in **1**. Assignment of the four aryl substituents in **1** was consistent with all of the observed HMBC correlations (Figure 2).

The structural fragments assembled thus far accounted for 20 of the 21 degrees of unsaturation in **1**. The last unsaturation equivalent was identified as an oxolane at the core of the molecule. The composition and connectivity of the central five-membered ring was confirmed by proton–proton coupling between H-3 (*δ*_H_ 4.09, d, *J* = 13.8 Hz) and H-4 (*δ*_H_ 3.91, d, *J* = 13.8 Hz) and HMBC correlations between H-3/C-26/C-27/C-2/C-16, H-4/C-32/C-33/C-5/C-6, H-27/C-3, H-31/C-3, H-33/C-4, and H-37/C-4. Finally, a methyl acetal (*δ*_H_ 3.40 3H, s) group at C-2 (*δ*_C_ 109.1) and a hemiacetal (*δ*_H_ 7.40 OH, s) group at C-5 (*δ*_C_ 102.3) were confirmed by HMBC correlations between 2-OCH_3_/C-2 and 5-OH/C-4/C-5/C-6. To verify the positions of the hemiacetal and acetal carbons, several long-range heteronuclear correlation NMR experiments were conducted including HMBC optimized for *J*_CH_ = 3.5 Hz or *J*_CH_ = 2.0 Hz, and an LR-HSQMBC experiment optimized for *J*_CH_ = 2.0 Hz [12]. As a result, we confirmed 4-bond correlations from H-27 and H-31 to C-2, as well as H-33 and H-37 to C-5 (Figure 1). This completed assignment of the planar structure of suberitamide A (**1**), which consists of a central oxolane ring containing hemiacetal and methyl acetal groups, with the *para*-hydroxystyrylamide carbonyls attached at C-2 and C-5, and the dihydroxy aromatic rings linked at C-3 and C-4.

The oxolane ring of **1** possessed four contiguous stereogenic centers at C-2, C-3, C-4, and C-5. The relative configuration of this type of five-membered ring system is often difficult to assign by conventional NMR analyses (NOEs, ^1^H-^1^H couplings) due to conformational flexibility and puckering of the ring [13]. While the 1,3-*cis* NOE effect is generally a reliable tool for assigning relative configuration in a system like this, NOEs between vicinal protons on five-membered rings are not necessarily diagnostic. A *J*-based approach to determine the relative configuration in substituted five-membered oxolane rings using the analysis of homonuclear and heteronuclear *J* couplings, and quantum mechanical DFT methods has recently been described [14,15]. It established specific ranges and thresholds, based on the magnitude of 2- and 3-bond H/C heteronuclear couplings, that defined the relative orientation of substituents around the ring system.

We initially measured selective 1D ROESY correlations with **1** and observed positive interactions between 5-OH/H-3, H-3/2-OCH_3_, and H-4/7-NH in DMSO-*d*_6_ (Figure 2A and Supporting Information). Thus, the configuration of the oxolane ring of **1** should have H-3/H-4-*trans* and 5-OH/H-3-*cis.* Support for the H-3/H-4-*trans* relationship, in which there is approximately a 180° dihedral angle between H-3 and H-4, was provided by comparing the H-3/H-4 coupling constant we measured (13.8 Hz) with that calculated using MestReJ (13.86 Hz) for a similar ^3^*J*_HH_ with a 180° dihedral angle (Figure 2B and Supporting Information) [16].

**Figure 3:**
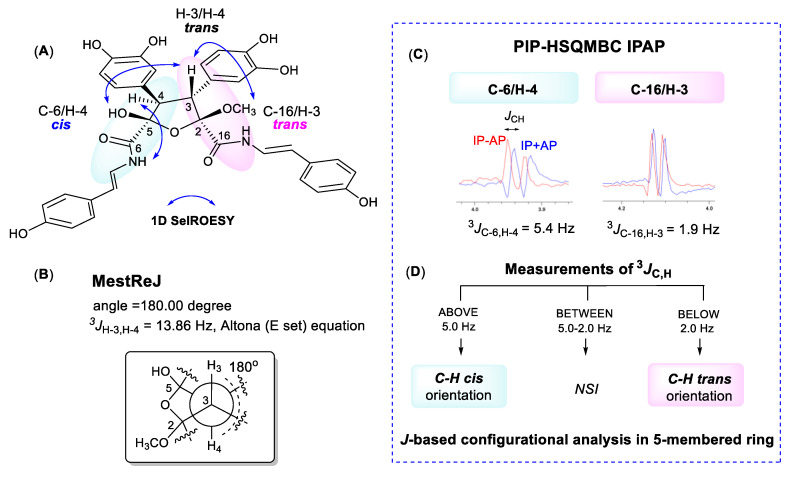
(**A**) Selective 1D ROESY correlations around the five-membered ring in **1**. (**B**) calculated proton–proton coupling when the H-3/H-4 dihedral angle is 180°. (**C**) 8.0 Hz optimized PIP-HSQMBC IPAP (pure in-phase heteronuclear single quantum multiple bond correlation in-phase and anti-phase) spectra were acquired. The in-phase plus anti-phase (IP + AP) and in-phase minus anti-phase (IP − AP) datasets were processed and corresponding 1D slices (blue, IP + AP; red, IP − AP) were overlaid at the C-3, C-4, C-6, and C-16 carbon chemical shifts. (**D**) flow chart to use ^3^*J*_C,H_ values for configurational analysis of five-membered oxolane ring (NSI: no stereochemical information can be extracted) [14,15].

For the *J*-based analysis, we measured the ^1^H-^13^C heteronuclear couplings using an HSQC-HECADE (heteronuclear couplings from ASSCI-domain experiments with E.COSY-type cross peaks) experiment (Supporting Information) [17,18]. This provided complimentary evidence for a *trans* orientation between H-3 and H-4 with characteristic large couplings measured for ^2^*J*_C3,H4_ (−7.1 Hz) and ^2^*J*_C4,H3_ (−9.6 Hz). However, with this experiment it was not possible to measure the desired ^3^*J*_C,H_ couplings between H-4/C-6 and H-3/C-16 because the carbons are nonprotonated. Thus, we employed a PIP (pure in-phase) HSQMBC IPAP experiment which allows accurate extraction of proton–carbon coupling constants, ^n^*J*_CH_ (n > 1) for nonprotonated carbons [11]. For suberitamide A (**1**), we initially acquired both the 8.0 Hz optimized IP (in-phase) and AP (anti-phase) PIP-HSQMBC spectra (Supplementary Materials, Figures S10–S13). Then, we applied the IPAP methodology which involves adding the in-phase and anti-phase components (IP + AP) or alternately subtracting the anti-phase component (IP − AP) of the two datasets. After executing the addition/subtraction steps, 1D slices were extracted from the IP + AP and IP − AP datasets at the frequencies of the carbons of interest. It was then possible to accurately measure the long-range proton carbon coupling constants from the overlaid *α*/*β* datasets (Figure 3C). A more-detailed description of this procedure can be found in the Supporting Information [19]. The heteronuclear *J* couplings measured in this experiment in DMSO-*d*_6_ were ^3^*J*_H−3,C−16_ = 1.9 Hz and ^3^*J*_H−4,C−6_ = 5.4 Hz. This *J*-based analysis confirmed that the substitution of the oxolane ring in **1** is H−4/C−6 *cis*, H−3/C−16 *trans*, and H-3/H-4 *trans* orientation (Figure 3D, Supporting Information). The relative configuration of **1** was thus determined to be 2R*, 3R*, 4R*, and 5S*. The absolute configuration of suberitamide A (**1**) could not be assigned by computational methods as there was no observable Cotton effect in the experimental ECD profile, and due to sidechain mobility and puckering of the central five-membered ring, 62 different conformers resulted from a conformational search using Macromodel software (version 12.0.012, Schrödinger Inc., New York, NY, USA).

**Figure 4:**
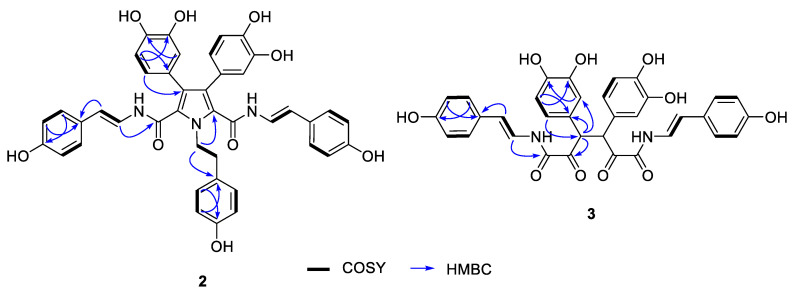
Selected 2D NMR correlations for suberitamides B (**2**) and C (**3**).

The molecular formula of suberitamide B (**2**) was established as C_42_H_35_N_3_O_9_ by HRESIMS analysis with 27 degrees of unsaturation, while observation of only half of the predicted signals in the ^1^H and ^13^C NMR spectra (Table 2) revealed it was a symmetrical molecule. Comparison with the NMR data for **1** showed there were appropriate signals for two symmetrical *para*-hydroxystyrylamide (*p*HSA) and 3,4-dihydroxyphenyl groups in **2**. In addition, the presence of a tyramine unit in **2** was indicated by a pair of aromatic doublets at *δ*_H_ 6.99 (2H, d, *J* = 8.4 Hz) and 6.68 (2H, d, *J* = 8.4 Hz) and a pair of broad triplets at *δ*_H_ 4.69 (2H, br t, *J* = 7.6 Hz) and 2.99 (2H, br t, *J* = 7.6 Hz) in MeOH-*d*_4_. The structural fragments assembled thus far accounted for 24 degrees of unsaturation, and only two pairs of equivalent sp^2^ carbons (*δ*_C_ 127.8 and 127.9) were left to account for the three remaining double bond equivalents. Considering the symmetry and molecular formula requirements, the remaining fragment was a fully substituted pyrrole ring formed with the nitrogen of the tyramine moiety. The structure of suberitamide B (**2**) was assigned with the two enamide carbonyls attached at C-2 and C-5 of the pyrrole, the two 3,4-dihydroxyphenyl rings substituted at C-3 and C-4, and a tyramine moiety with the nitrogen incorporated into the central pyrrole ring. This assignment was supported by HMBC correlations from H-37 to C-2/C-5, H-27/H-31 to C-3, and H-33/37 to C-4 (Figure 4). Suberitamide B (**2**) is structurally related to the storniamides, which are nonsymmetrical pyrrole-containing aryl alkaloids reported from a *Cliona* sp. marine sponge collected in Patagonia [20].

The molecular formula of suberitamide C (**3**) was established as C_34_H_28_N_2_O_10_ by HRESIMS measurements, with 22 degrees of unsaturation. Detailed examination of the ^1^H and ^13^C NMR data, aided by 2D NMR experiments, revealed this compound was also a symmetrical molecule. Characteristic NMR signals for *para*-hydroxystyrylamide and 3,4-dihydroxyphenyl groups were apparent, as well as an aliphatic methine (*δ*_H_ 5.15/*δ*_C_ 55.5) and a ketone carbonyl (*δ*_C_ 197.2). HMBC correlations from the methine proton linked the methine group to a 3,4-dihydroxyphenyl moiety and to the ketone carbon (Figure 4). This suggested that suberitamide C (**3**) was a symmetrical, ring-opened homologue where the oxolane ring of **1** was opened and the C-2 and C-5 carbons were both ketones. An HMBC correlation from the aliphatic methine proton to the methine carbon supported the conclusion that two symmetrical subunits of **3** were linked via the methine carbons. The configuration of the methine carbons in suberitamide C (**3**) was assigned by analogy with **1**. Consistent with this assumption of either 3*R*, 4*R* or 3*S*, 4*S* stereochemistry, compound **3** was optically active ([α]25D +86.7), while a meso form (3*R*, 4*S* or 3*S*, 4*R*) of **3** would be achiral. The *α*-ketoenamide functionality found in suberitamide C (**3**) is an unusual structural feature for a natural product, and while a close relationship between **1** and **3** is apparent, compound **1** was stable and no conversion of **1** to **3** was observed over an extended period of time and in variety of solvents.

**Figure 5:**
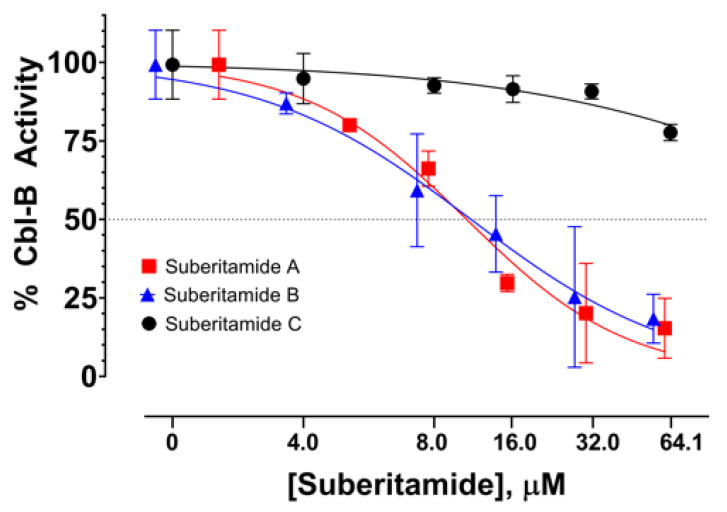
Dose-response curves of Cbl-b inhibitory activity for suberitamides A–C (**1**–**3**).

Suberitamides A–C (**1**–**3**) were tested for their ability to inhibit autoubiquitination of the E3 ubiquitin ligase Cbl-b [10]. Compounds **1** and **2**, with cyclic central cores comprised of oxolane and pyrrole rings, respectively, abrogated Cbl-b enzymatic activity with low micromolar potencies. The EC_50_ value for both **1** and **2** was approximately 11 µM, while the ring-opened homologue **3** was inactive at a high-test concentration of 64 µM (Figure 5). This suggests that the structural rigidity imparted by the cyclized core of **1** and **2** is necessary for activity, since the more flexible, ring-opened derivative **3** is inactive. Cbl-b functions as a negative regulator of immune activation, thus it represents an attractive potential target for immune system modulation. Inhibitors of Cbl-b could have therapeutic applications such as enhancing the innate anticancer immune response. While several natural products with Cbl-b inhibitory properties have been reported, they generally are cationic metabolites with quaternary amine groups [10]. Suberitamides A (**1**) and B (**2**), which do not have fixed positive charges, represent a new class of Cbl-b inhibitors that could provide a structural framework for further therapeutic development.

## 3. Materials and Methods

### 3.1. General Experimental Procedures

Optical rotations were measured on a Rudolph research analytical AUTOPOL IV automatic polarimeter (Rudolph Research Analytical, Hackettstown, NJ, USA), IR spectra were recorded with a Bruker ALPHA II FT-IR spectrometer (Bruker, Billerica, MA, USA), and UV spectra were measured with a Thermo Scientific Nanodrop 2000C spectrophotometer (Thermo Fisher Scientific, Waltham, MA, USA). High-performance liquid chromatography (HPLC) was performed using a Varian ProStar 215 solvent delivery module equipped with a Varian Prostar 320 UV-Vis detector (Agilent Technologies, Santa Clara, CA, USA), operating under Star 6.41 chromatography workstation software (6.41, Agilent Technologies, Santa Clara, CA, USA). NMR spectra were obtained with a Bruker Avance III NMR spectrometer (Bruker, Billerica, MA, USA) equipped with a 3 mm cryogenic probe and operating at 600 MHz for ^1^H and 150 MHz for ^13^C. Spectra were calibrated to residual solvent signals at *δ*_H_ 2.50 and *δ*_C_ 39.5 (DMSO-*d*_6_), and *δ*_H_ 3.31 and *δ*_C_ 49.0 (CD_3_OD). All 2D NMR experiments were acquired with nonuniform sampling (NUS) set to 50% or 25%. HMBC experiments were run with ^n^*J*_CH_ = 8.0, 3.5, or 2.0 Hz, and the LR-HSQMBC experiment was optimized for ^n^*J*_CH_ = 2.0 Hz. HRESIMS data were acquired on an Agilent Technology 6530 Accurate-mass Q-TOF LC/MS (Agilent Technologies, Santa Clara, CA, USA).

### 3.2. Animal Material

Samples of the marine sponge *Pseudosuberites* sp. were collected by scuba at a depth of ~2.5 m from a hard substrate at Harkers Island, North Carolina, USA, and kept frozen until extraction. The collection was carried out by Sea Samples, for the Coral Reef Research Foundation, under contract with the National Products Branch, U.S. National Cancer Institute. A voucher specimen (voucher ID # 0YYD 3440) was deposited at the Smithsonian Institution, Washington, DC, USA.

### 3.3. Extraction and Isolation 

The animal material (442 g wet wt.) was ground and processed using the standard NCI method for marine samples to provide 17.2 g of organic solvent extract (NSC C032671) [21]. A 5.1 g aliquot of the *Pseudosuberites* sp. organic extract was subjected to C_18_ reversed-phase flash column chromatography using a step gradient elution with 100% hexane (fraction A, 195 mg), 100% CH_2_Cl_2_ (fraction B, 647 mg), 100% EtOAc (fraction C, 274 mg), 100% acetone (fraction D, 590 mg), and 100% MeOH (fraction E, 2.96 g). The active fraction C was separated by preparative reversed-phase HPLC (Agilent Dynamax C18 column, 21.4 mm × 250 mm, Santa Clara, CA, USA) eluting at 9 mL/min with a CH_3_CN–H_2_O gradient (20:80–60:40), yielding (*t*_R_ = 62 min) compound **2** (2.9 mg), as an amorphous oil. Further purification of the sub-fractions (11 and 12) by semi-preparative reversed-phase HPLC (Phenomenex Luna C18 column, 10 mm × 250 mm, Torrance, CA, USA) eluting at 3.0 mL/min with a CH_3_CN–H_2_O gradient (28:72–50:50), *t*_R_ = 51 min, and CH_3_CN–H_2_O gradient (35:65–65:35), *t*_R_ = 23 min, afforded compounds **3** (0.7 mg) and **1** (3.4 mg), respectively, as amorphous powders.

*Suberitamide A* (**1**): yellow, amorphous powder; [α]25D +7.5 (*c* 0.11, MeOH); UV (MeOH) *λ*_max_ (log *ε*) 215 (3.86), 291 (3.84) nm; IR (film) *ν*_max_ 3267, 1652, 1609, 1509, 1233, 1022 cm^-1^; ^1^H and ^13^C NMR, Table 1; HRESIMS *m*/*z* 657.2090 [M + H]^+^ (calcd for C_35_H_33_N_2_O_11_, 657.2084)

*Suberitamide B* (**2**): yellow, amorphous powder; UV (MeOH) *λ*_max_ (log *ε*) 217 (4.19), 292 (3.96), 337 (3.97) nm; IR (film) *ν*_max_ 3339, 1649, 1608, 1503, 1240, 1200 cm^−1^; ^1^H and ^13^C NMR, Table 2; HRESIMS *m*/*z* 726.2447 [M + H]^+^ (calcd for C_42_H_36_N_3_O_9_, 726.2452)

*Suberitamide C* (**3**): yellow, amorphous powder; [*α*]25D + 86.7 (*c* 0.06, MeOH); UV (MeOH) *λ*_max_ (log *ε*) 217 (3.48), 288 (3.44) nm; IR (film) *ν*_max_ 3300, 1677, 1608, 1512, 1207, 1139 cm^−1^; ^1^H and ^13^C NMR, Table 2; HRESIMS *m*/*z* 625.1808 [M + H]^+^ (calcd for C_34_H_29_N_2_O_10_, 625.1820)

### 3.4. Calculation of Dihedral Bond Angles in MestReJ

Calculation of the dihedral angle between H-3 and H-4 was performed using the Altona equation with 4 substituents at the C-3 and C-4 positions. The resulting ^3^*J*_H−3,H−4_ value for a 180° dihedral angel was 13.9 Hz [16].

### 3.5. NMR Measurements

The HSQC-HECADE (heteronuclear couplings from ASSCI-domain experiments with E.COSY-type cross peaks) experiment was recorded using DIPSI (decoupling in the presence of scalar interactions) during the 40 ms isotropic mixing period, using a bandwidth of 10 kHz and a *J*-scale factor of 1. Prior to Fourier transformation, zero filling was performed to expand the data to at least double the number of acquired data points. The PIP–HSQMBC spectra were recorded with an inter-pulse delay optimized to 8 Hz (Δ = 1/2 × ^n^*J*_CH_ = 62.5 ms). The recycle delay was 1 s and 256 scans were collected for each of the 64 t_1_ increments, with 4096 data points in each t_1_ increment. Prior to Fourier transformation, zero-filling to 1024 points in F1, 8192 points in F2 and a squared sine-bell apodization phase-shifted 90° in both dimensions was applied. The final digital resolution along the detected F2 dimension was of 0.4 Hz. The total experimental time was about 6 h 22 min 53 s for each IP and AP dataset. For the IPAP technique, IP and AP-HSQMBC datasets were separately recorded and then added/subtracted in the time-domain to provide two separate α/β data sets. Additional fitting processes, simulations, and experimental spectra are available in the Supporting Information.

### 3.6. Cbl-b Biochemical Assay

Details of the Cbl-b assay system have been described previously [10]. In brief, dose response experiments with the purified compounds were carried out in Tris-HCl buffer (pH 7.5) that contained 15 nM E1 protein (UBE1), 75 nM E2 protein (UBC-H5) [22], 112 nM Cbl-b protein (N1/2 Construct) [23], 75 nM biotinylated ubiquitin, 750 nM ubiquitin, 0.1 mM dithiothreitol, 0.5 mg/mL bovine gelatin type B, 0.5 mM magnesium chloride, and 0.01% Triton X-100. Addition of ATP into the enzyme solution initiated the enzymatic reaction cascade. Initiated reactions were then transferred to plates that had been precoated overnight with 10 µg/mL of the polyubiquitin binding portion of Cbl-b (UBA) [24] which allowed for the binding and specific enrichment of autopolyubiquitinated Cbl-b. After 1 h, the reactions were quenched and the reaction plates were sealed and incubated overnight at room temperature. The following day, reaction plates were probed with avidin-conjugated horse radish peroxidase, washed three times, and then an avidin-HRP dependent fluorescent signal (indicating the presence of avidin-HRP/biotin-polyubiquitin complexes bound by the UBA-coated plate) was detected (excitation 325 nm, emission 420 nm) using a Tecan Infinite M1000 plate reader (Tecan, Zürich, Switzerland) Fluorescence data for each test well was background corrected and normalized to its vehicle control according to the following formula:

$$\%\;Activity=\;\frac{\left(Substance\;RFU-DMSO\;Control_{No\;ATP}\right)}{\left(DMSO\;Control_{+ATP}-DMSO\;Control_{No\;ATP}\right)}*100$$

## 4. Conclusions

Chemical investigation of the sponge *Pseudosuberites* sp. provided three new aryl alkaloids named suberitamides A–C (**1**–**3**). Structure elucidation of suberitamide A (**1**) was aided by the application of new NMR methodologies including the PIP HSQMBC IPAP experiment [11] for measuring key long-range heteronuclear (C, H) coupling constants. This allowed unambiguous assignment of the relative configuration of the four contiguous stereogenic centers in the flexible oxolane ring of **1**. Suberitamides B (**2**) and C (**3**) are structurally related symmetrical molecules and **3** possesses *α*-ketoenamide functionalities, which are rare in natural products. In a biochemical assay for inhibitors of the ubiquitin ligase Cbl-b, suberitamides A (**1**) and B (**2**), with aryl-substituted oxolane and pyrrole central rings, respectively, were active at low micromolar concentrations, while the ring-opened derivative **3** was inactive. The suberitamides represent a new class of natural product inhibitors of Cbl-b ubiquitin ligase activity that are structurally distinct from other known inhibitors of this enzyme.

## Figures and Tables

**Table 1:** ^13^C NMR (150 MHz) and ^1^H NMR (600 MHz) Data for Suberitamide A (**1**).

Position	*δ*_C_ (type) *^a^*	*δ*_H_ (J in Hz) *^a^*	*δ*_C_ (type) *^b^*	*δ*_H_ (J in Hz) *^b^*
2	109.1 C		110.9 C	
2-OCH_3_	51.6, CH_3_	3.40, s	52.6, CH_3_	3.52, s
3	53.3, CH	4.09, d (13.8)	55.4, CH	4.22, d (13.8)
4	54.6, CH	3.91, d (13.8)	56.3, CH	4.04, d (13.8)
5	102.3 C		103.8 C	
5-OH		7.40, s		
6	169.4, s		171.6, s	
7-NH		10.52, d (10.2)		
8	120.2, CH	7.29, dd (14.6, 10.2)	120.7, CH	7.37, d (14.6)
9	114.8, CH	6.43, d (14.6)	117.2, CH	6.40, d (14.6)
10	126.9, C		128.9, C	
11, 15	126.8, CH	7.24, d (8.4)	128.0, CH	7.25, d (8.4)
12, 14	115.6, CH	6.72, d (8.4)	116.6, CH	6.75, d (8.4)
13	156.4, C		157.8, C	
13-OH		9.47, s		
16	166.0, C		168.6, C	
17-NH		10.50, d (10.2)		
18	120.1, CH	7.04, dd (14.6, 10.2)	120.5, CH	7.10, d (14.6)
19	114.3, CH	6.29, d (14.6)	117.1, CH	6.35, d (14.6)
20	126.9, C		128.8, C	
21, 25	126.7, CH	7.18, d (8.4)	128.0, CH	7.20, d (8.4)
22, 24	115.6, CH	6.70, d (8.4)	116.5, CH	6.73, d (8.4)
	156.3, C		157.7, C	
23-OH		9.44, s		
26	124.8, C		126.5, C	
27	115.8, CH	6.52, d (1.8)	116.7, CH	6.66, d (1.8)
28	144.4, C		146.1, C	
28-OH		8.71, s		
29	144.5, C		145.8, C	
29-OH		8.74, s		
30	115.0, CH	6.50, d (8.4)	115.9, CH	6.60, d (8.4)
31	119.2, CH	6.36, dd (8.4, 1.8)	121.2, CH	6.54, dd (8.4, 1.8)
32	125.0, C		126.6, C	
33	116.6, CH	6.55, d (1.8)	117.5, CH	6.69, d (1.8)
34	144.7, C		145.7, C	
34-OH		8.68, s		
35	144.6, C		145.9, C	
35-OH		8.69, s		
36	115.2, CH	6.51, d (8.4)	116.2, CH	6.61, d (8.4)
37	120.1, CH	6.38, dd (8.4, 1.8)	122.1, CH	6.52, dd (8.4, 1.8)

*^a,b^*NMR spectra were acquired in a DMSO-*d*_6_ and CD_3_OD, respectively.

**Table 2:** ^13^C NMR (150 MHz) and ^1^H NMR (600 MHz) Data for suberitamides B (**2**) and C (**3**) in CD_3_OD.

	2		3	
Position	*δ*_C_ (type)	*δ*_H_ (*J* in Hz)	*δ*_C_ (type)	*δ*_H_ (*J* in Hz)
2, 5	127.9, C		197.2, C	
3, 4	127.8, C		55.5, CH	5.15, s
6, 16	161.1, C		159.3, C	
8, 18	120.5, CH	7.27, d (14.6)	120.0, CH	7.14, d (14.6)
9, 19	115.6, CH	5.71, d (14.6)	118.6, CH	6.42, d (14.6)
10, 20	128.8, C		128.7, C	
11,15,21,25	127.8, CH	7.11, d (8.4)	128.1, CH	7.18, d (8.4)
12,14,22,24	116.5, CH	6.69, d (8.4)	116.5, CH	6.70, d (8.4)
13, 23	157.7, C		158.1, C	
26, 32	126.3, C		125.8, C	
27, 33	118.6, CH	6.61, d (1.8)	117.7, CH	6.53, d (1.6)
28, 34	146.3, C		146.5, C	
29, 35	145.9, C		146.1, C	
30, 36	116.4, CH	6.74, d (8.4)	116.5, CH	6.60, d (8.4)
31, 37	123.2, CH	6.53, dd (8.4, 1.8)	122.1, CH	6.42, dd (8.4, 1.6)
38	49.3, CH_2_	4.69, br t (7.6)		
39	38.7, CH_2_	2.99, br t (7.6)		
40	130.5, C			
41, 45	131.2, CH	6.99, d (8.4)		
42, 44	116.2, CH	6.68, d (8.4)		
43	157.2, C			

## Supplementary Materials

The following are available online at https://www.mdpi.com/1660-3397/18/11/536/s1, Experimental procedures, additional figures, and full spectroscopic data for compounds **1**–**3**.
