# Supplementary material for: Suberitamides A–C, Aryl Alkaloids from a Pseudosuberites sp. Marine Sponge that Inhibit Cbl-b Ubiquitin Ligase Activity
Source: Mar Drugs. 2020 Oct 28;18(11):536. doi: 10.3390/md18110536 (PMC7693676; doi:10.3390/md18110536)
Supplement: Supplementary file 1 [file marinedrugs-18-00536-s001.pdf]

Suberitamides A-C, Aryl Alkaloids from a *Pseudosuberites* sp. Marine Sponge that Inhibit Cbl-b Ubiquitin Ligase Activity

Chang-Kwon Kim,[1] Dongdong Wang,[1] Brice A. P. Wilson,[1] Josep Saurí,[2] Donna Voeller,[3] Stanley Lipkowitz,[3] Barry R. O'Keefe,[1,4] and Kirk R. Gustafson[1,*]

[1] Molecular Targets Program, Center for Cancer Research, National Cancer Institute, Frederick, Maryland 21702-1201, USA

National Laboratory for Cancer Research, Frederick, Maryland 21702-1201, United States

[2] Structure Elucidation Group, Analytical Research and Development, Merck & Co., Inc., Boston, Massachusetts, 02115, USA

[3] Women's Malignancies Branch, Center for Cancer Research, National Cancer Institute, Bethesda, Maryland 20892-1578, USA

[4] Natural Products Branch, Developmental Therapeutics Program, Division of Cancer Treatment and Diagnosis, National Cancer Institute, Frederick, Maryland 21701-1201, USA

[*] Correspondence: gustafki@mail.nih.gov

Contents

Table S1. ^{13}C NMR (150 MHz) and ^{1}H NMR Data (600 MHz) of Compound **1** in DMSO-d_6 S4
Figure S1. ^{1}H NMR (600 MHz, DMSO-d_6) spectrum of **1** S5
Figure S2. ^{13}C NMR (150 MHz, DMSO-d_6) spectrum of **1** S5
Figure S3. Edited HSQC NMR (DMSO-d_6) spectrum of **1** S6
Figure S4. COSY NMR (DMSO-d_6) spectrum of **1** S6
Figure S5. HMBC NMR (DMSO-d_6) spectrum of **1** S7
Figure S6. ESI-HR MS data of **1** S7
Figure S7. IR & UV spectra of **1** S8
Figure S8. 1D-Selective ROESY NMR (DMSO-d_6) spectra of **1** S9
Figure S9. Calculated dihedral angle along the C-3/C-4 bond using MestReJ v1.1 and selective ^{1}H NMR (600 MHz, DMSO-d_6) spectrum of **1** S10
Figure S10. IP PIP-HSQMBC NMR (*cnst* 25 = 0, DMSO-d_6) spectrum of **1** S11
Figure S11. Expanded IP PIP-HSQMBC NMR spectrum of **1** S11
Figure S12. AP PIP-HSQMBC NMR (*cnst* 25 = 1, DMSO-d_6) spectrum of **1** S12
Figure S13. Expanded AP PIP-HSQMBC NMR spectrum of **1** S12
Figure S14. IPAP: addition/subtraction procedure using “adsu” in TopSpin 4.0.7 S13
Figure S15. Extracted 1D carbon slices (600 MHz, DMSO-d_6) spectra of **1** S17
Figure S16. Flow chart to use $^{3}J_{C,H}$ values for configurational analysis of five-membered rings S18
Figure S17. ^{1}H NMR (600 MHz, MeOH-d_4) spectrum of **1** S19
Figure S18. ^{13}C NMR (150 MHz, MeOH-d_4) spectrum of **1** S19
Figure S19. Edited HSQC NMR (MeOH-d_4) spectrum of **1** S20
Figure S20. COSY NMR (MeOH-d_4) spectrum of **1** S20
Figure S21. HMBC NMR (MeOH-d_4) spectrum of **1** S21
Figure S22. LR-HSQMBC NMR (MeOH-d_4) spectrum of **1** S21
Figure S23. HMBC NMR (J_{CH} = 3.5 Hz, MeOH-d_4) spectrum of **1** S22
Figure S24. HMBC NMR (J_{CH} = 2.0 Hz, MeOH-d_4) spectrum of **1** S22
Figure S25. HSQC-HECADE NMR (MeOH-d_4) spectrum of **1** S23
Figure S26. Expanded HSQC-HECADE NMR (MeOH-d_4) spectrum of **1** S23
Figure S27. ^{1}H NMR (600 MHz, MeOH-d_4) spectrum of **2** S24
Figure S28. ^{13}C NMR (150 MHz, MeOH-d_4) spectrum of **2** S24
Figure S29. Edited HSQC NMR (MeOH-d_4) spectrum of **2** S25
Figure S30. COSY NMR (MeOH-d_4) spectrum of **2** S25
Figure S31. HMBC NMR (MeOH-d_4) spectrum of **2** S26
Figure S32. ESI-HR MS data of **2** S26
Figure S33. IR & UV spectra of **2** S27

Figure S34. ^{1}H NMR (600 MHz, MeOH-d_4) spectrum of **3** S28
Figure S35. ^{13}C NMR (150 MHz, MeOH-d_4) spectrum of **3** S28
Figure S36. Edited HSQC NMR (MeOH-d_4) spectrum of **3** S29
Figure S37. COSY NMR (MeOH-d_4) spectrum of **3** S29
Figure S38. HMBC NMR (MeOH-d_4) spectrum of **3** S30
Figure S39. ESI-HR MS data of **3** S30
Figure S40. IR & UV spectra of **3** S31
Figure S41. Relative configuration of compound **3** S32
Figure S42. Dose-response curves of Cbl-b inhibitory activity for **1-3** S33

Table S1. ^{13}C **NMR (150 MHz) and** ^{1}H **NMR Data (600 MHz) of Compound 1 in DMSO-*d*$_6$.**

Position	δ_C (type)	δ_H (*J* in Hz)	HMBC
2	109.1, C		
2-OCH_3	51.6, CH_3	3.40, s	2
3	53.3, CH	4.09, d (13.8)	2, 4, 16, 26, 27, 31
4	54.6, CH	3.91, d (13.8)	3, 5, 6, 32, 33, 37
5	102.3, C		
5-OH		7.40, s	4, 5, 6
6	169.4, C		
7-NH		10.52, d (10.2)	6
8	120.2, CH	7.29, dd (14.6, 10.2)	6, 9, 10
9	114.8, CH	6.43, d (14.6)	8, 10, 11, 15
10	126.9, C		
11, 15	126.8, CH	7.24, d (8.4)	9, 11, 13, 15
12, 14	115.6, CH	6.72, d (8.4)	10, 12, 13, 14
13	156.4, C		
13-OH		9.47, s	12, 13, 14
16	166.0, C		
17-NH		10.50, d (10.2)	
18	120.1, CH	7.04, dd (14.6, 10.2)	16, 19, 20
19	114.3, CH	6.29, d (14.6)	18, 20, 21, 25
20	126.9, C		
21, 25	126.7, CH	7.18, d (8.4)	19, 21, 23, 25
22, 24	115.6, CH	6.70, d (8.4)	20, 22, 23, 24
23	156.3, C		
23-OH		9.44, s	22, 23, 24
26	124.8, C		
27	115.8, CH	6.52, d (1.8)	2[a], 3, 29, 31
28	144.4, C		
28-OH		8.71, s	27, 28, 29
29	144.5, C		
29-OH		8.74, s	28, 29, 30
30	115.0, CH	6.50, d (8.4)	26, 28
31	119.2, CH	6.36, d (8.4, 1.8)	2[a], 3, 27, 29
32	125.0, C		
33	116.6, CH	6.55, d (1.8)	4, 5[a], 35, 37
34	144.7, C		
34-OH		8.68, s	33, 34, 35
35	144.6, C		
35-OH		8.69, s	34, 35, 36
36	115.2, CH	6.51, d (8.4)	32, 34
37	120.1, CH	6.38, dd (8.4, 1.8)	4, 5[a], 33, 35

[a]J_{CH}= 2 Hz optimized HMBC experiment

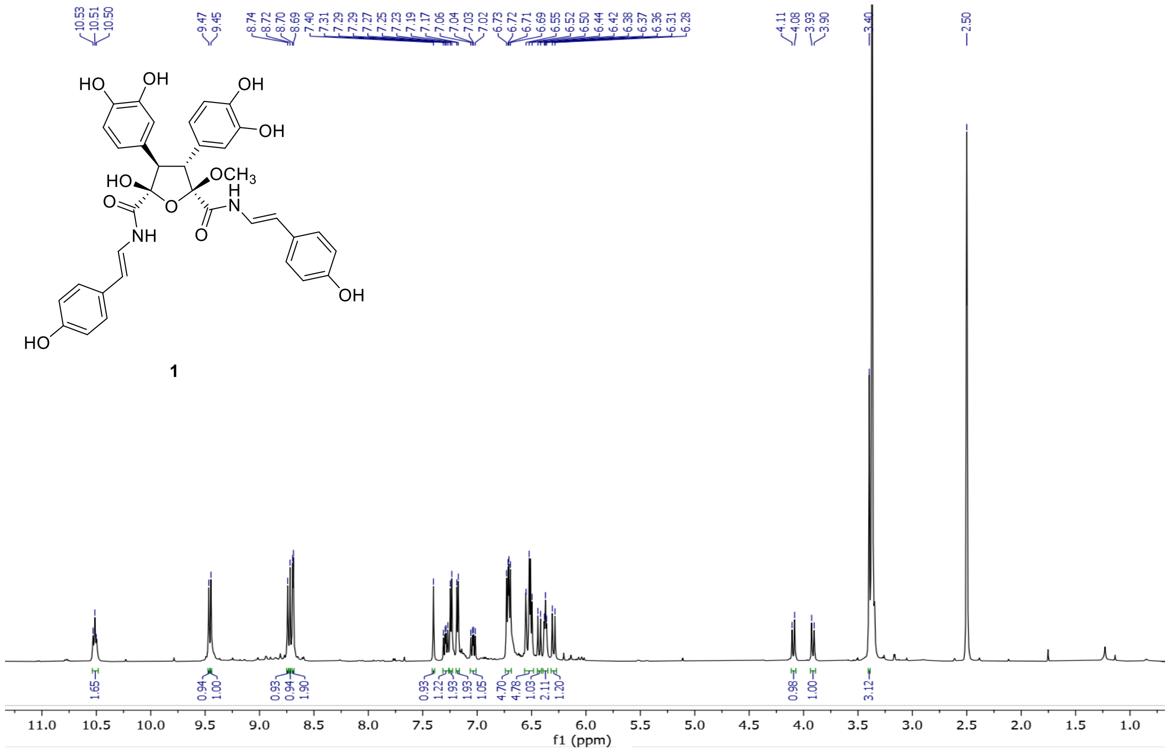

Figure S1. ^{1}H NMR (600 MHz, DMSO-d_6) spectrum of **1**

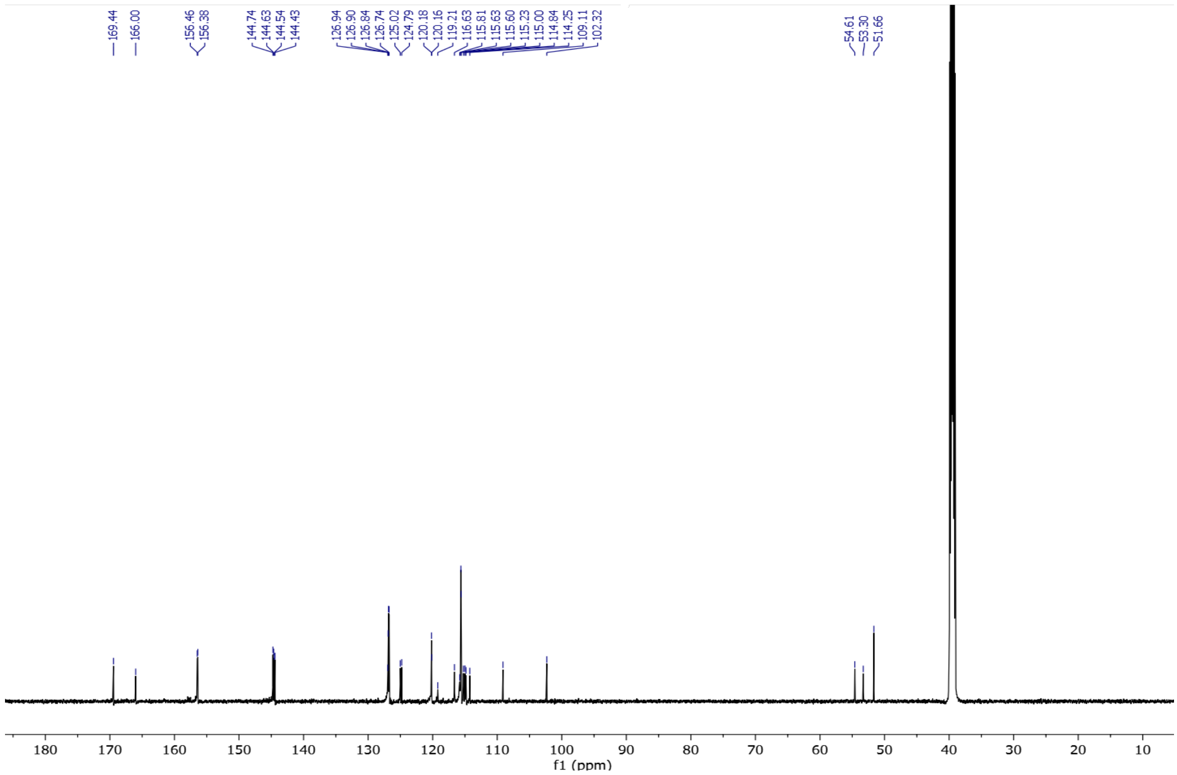

Figure S2. ^{13}C NMR (150 MHz, DMSO-d_6) spectrum of **1**

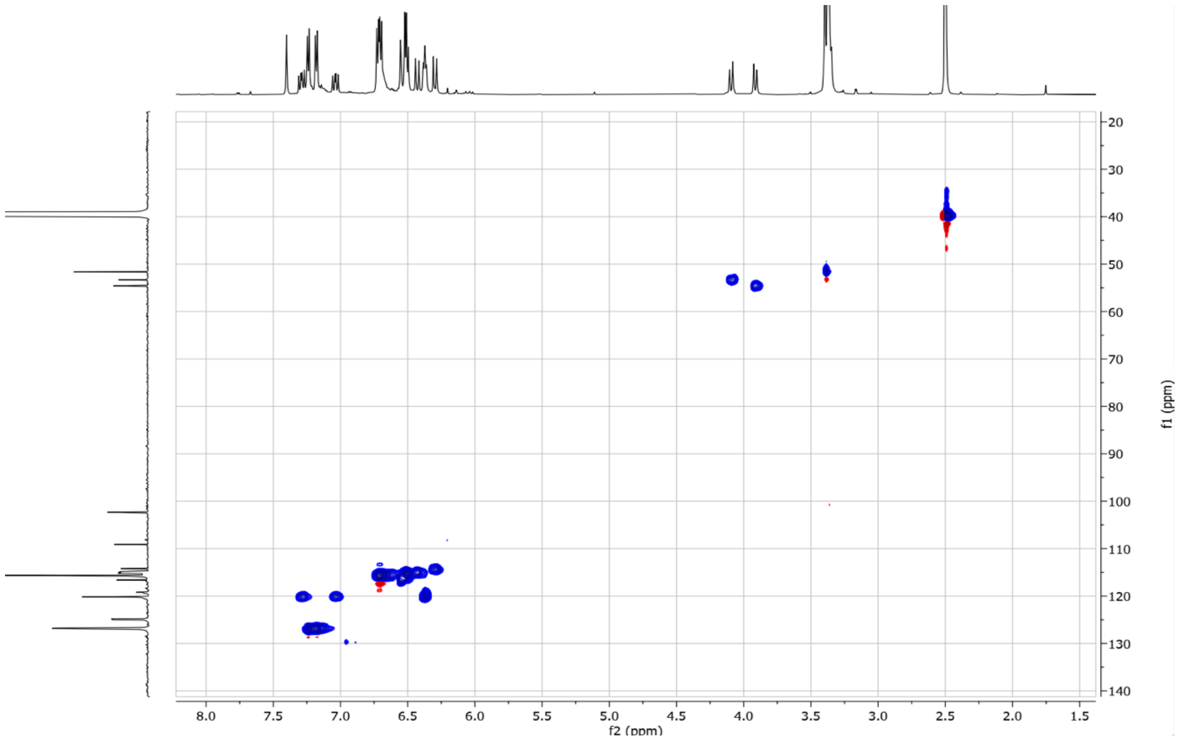

Figure S3. Edited HSQC NMR (DMSO-*d*$_6$) spectrum of **1**

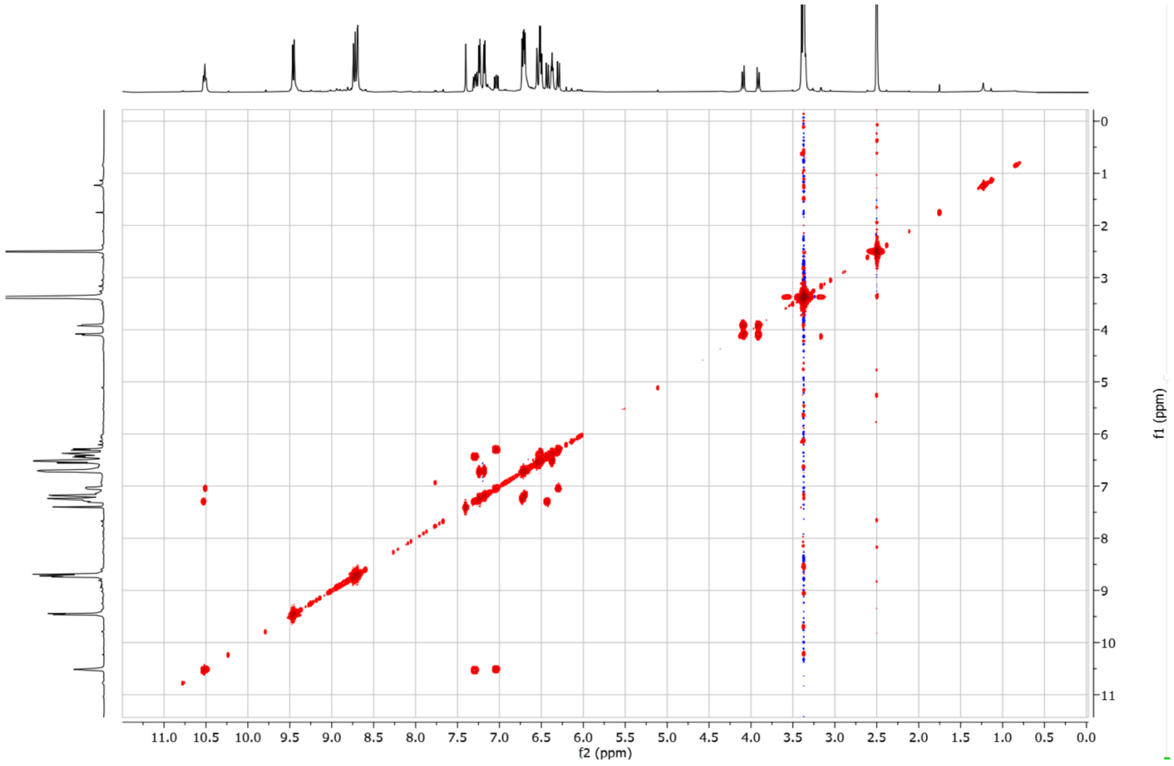

Figure S4. COSY NMR (600 MHz, DMSO-*d*$_6$) spectrum of **1**

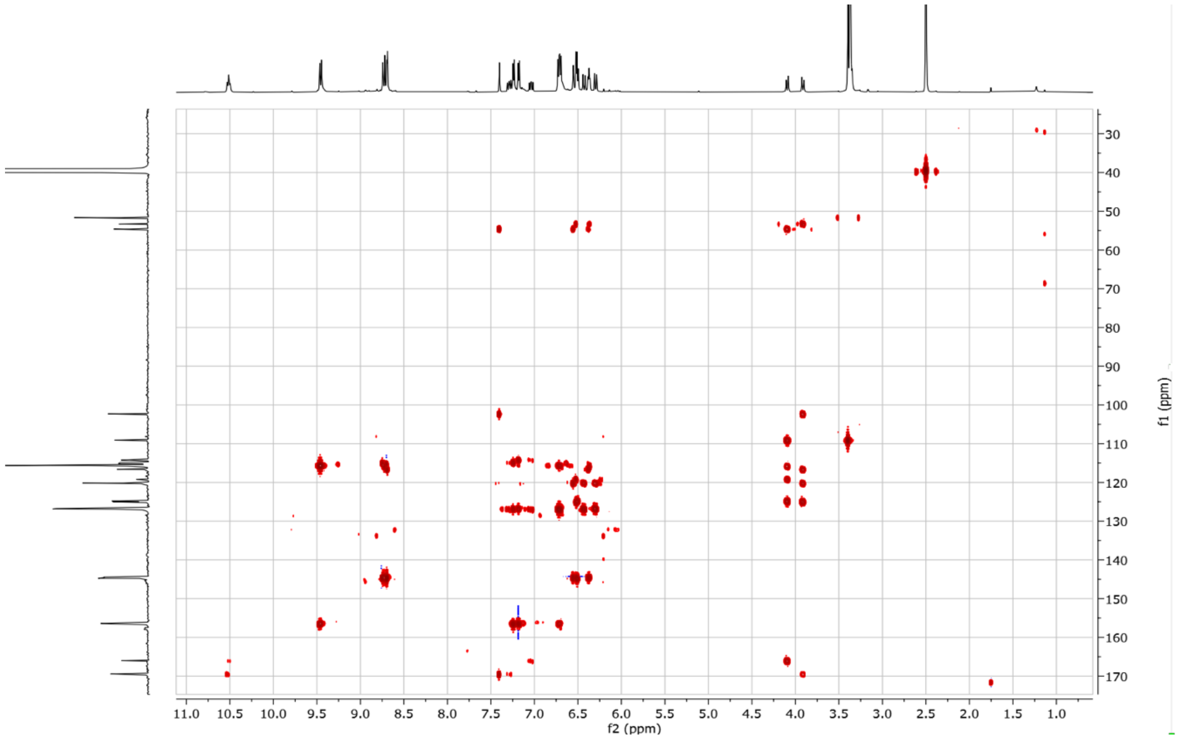

Figure S5. HMBC NMR (DMSO-d_6) spectrum of **1**

Analysis Report

Agilent | Trusted Answers

Sample Information

**Name**	176B112A_dil	**Data File Path**	C:\Users\Public\Desktop\QTOF data\Data\Chang\100819\176B112A_dil.d
**Sample ID**		**Acq. Time (Local)**	10/8/2019 10:04:07 AM (UTC-04:00)
**Instrument**	Instrument 1	**Method Path (Acq)**	D:\MassHunter\Methods\FIA_SM_LowFlow.m
**MS Type**	QTOF	**Version (Acq SW)**	6200 series TOF/6500 series Q-TOF B.09.00 (B9044.1 SP1)
**Inj. Vol. (ul)**	1	**IRM Status**	Some ions missed
**Position**	Vial 18	**Method Path (DA)**	
**Plate Pos.**		**Target Source Path**	
**Operator**		**Result Summary**	

Sample Spectra

+ Scan (rt: 0.366-1.012 min) **C35 H32 N2 O11**

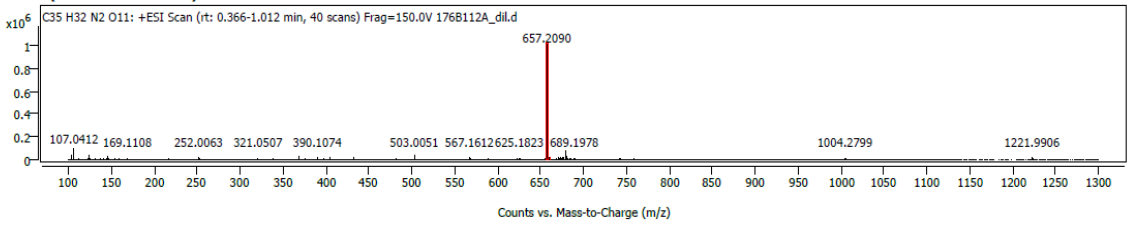

Spectrum Identification Table

Best ID Source	Name	Formula	Species	m/z	Diff (ppm)	CAS	Score	Score (Lib)	Score (DB)	Score (MFG)	Lib/DB
Yes MFG		C35 H32 N2 O11	(M+H)+	657.2090	1.49		96.53			96.53	

Figure S6. ESI-HR MS data of **1**

IR Spectrum Report

Instrument type	Alpha II
Accessory	ATR platinum Diamond 1 Refl #24C8F5322D
Spectrum file name	176B112A.0
Spectrum file path	T:\Data\MEAS\Chang
Measurement date and time	15/10/2019 22:20:54 (GMT-7)
Sample name	176B112A
Sample form	Instrument type and / or accessory

Spectrum

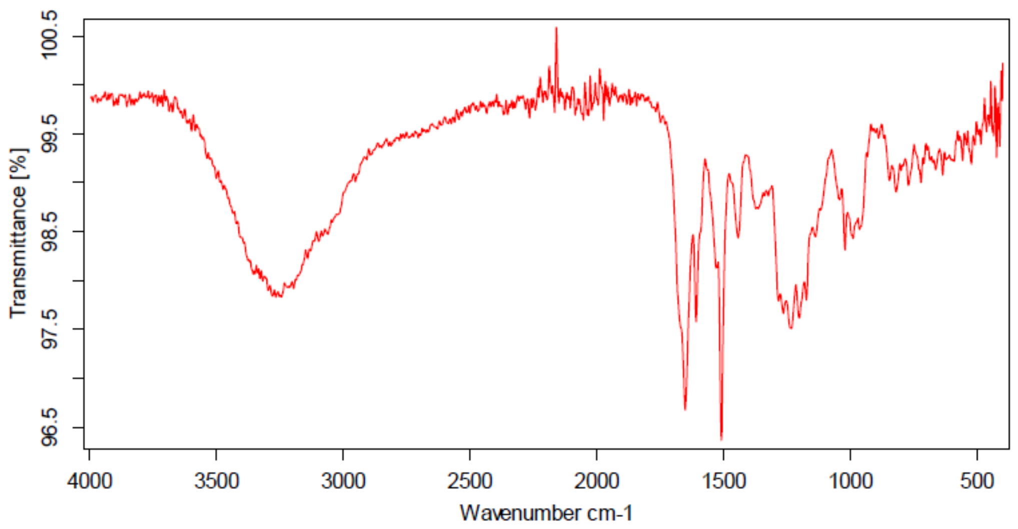

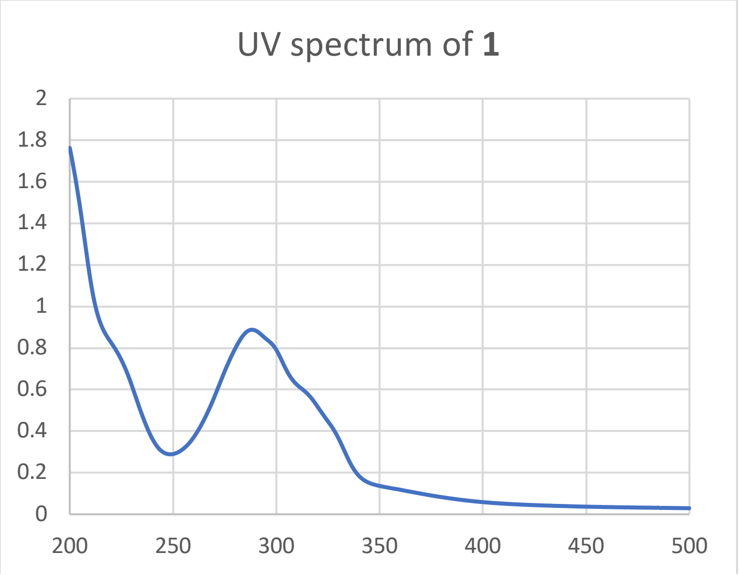

Figure S7. IR & UV spectra of **1**

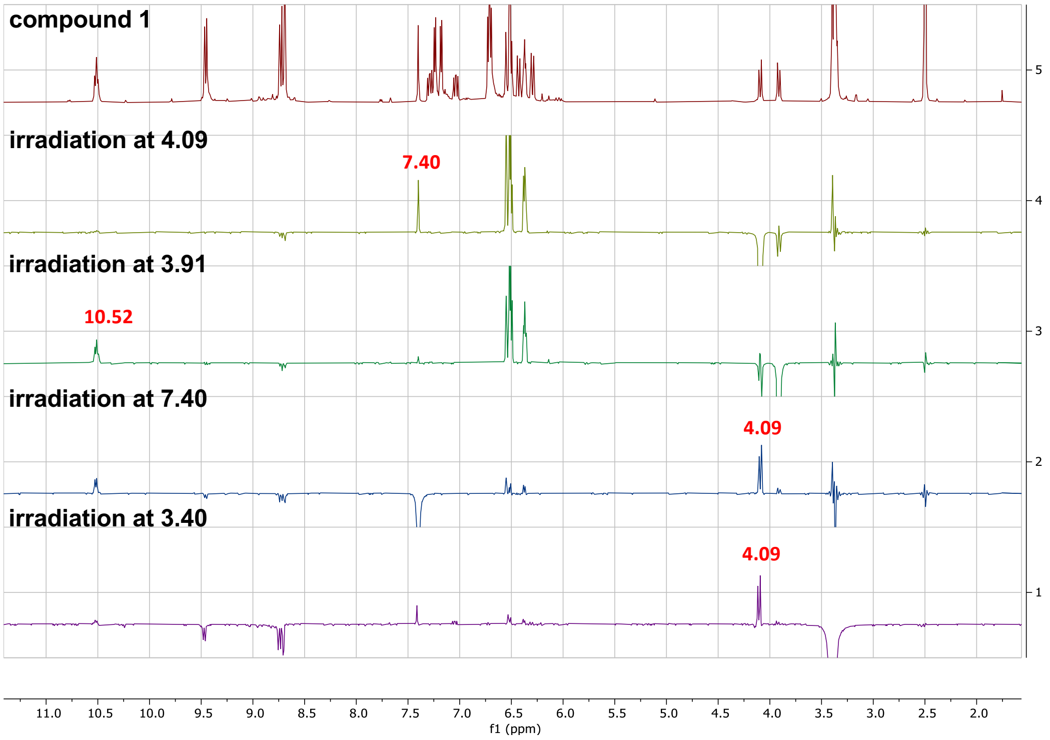

Figure S8. 1D-Selective ROESY NMR (600 MHz, DMSO-d_6) spectra of **1**

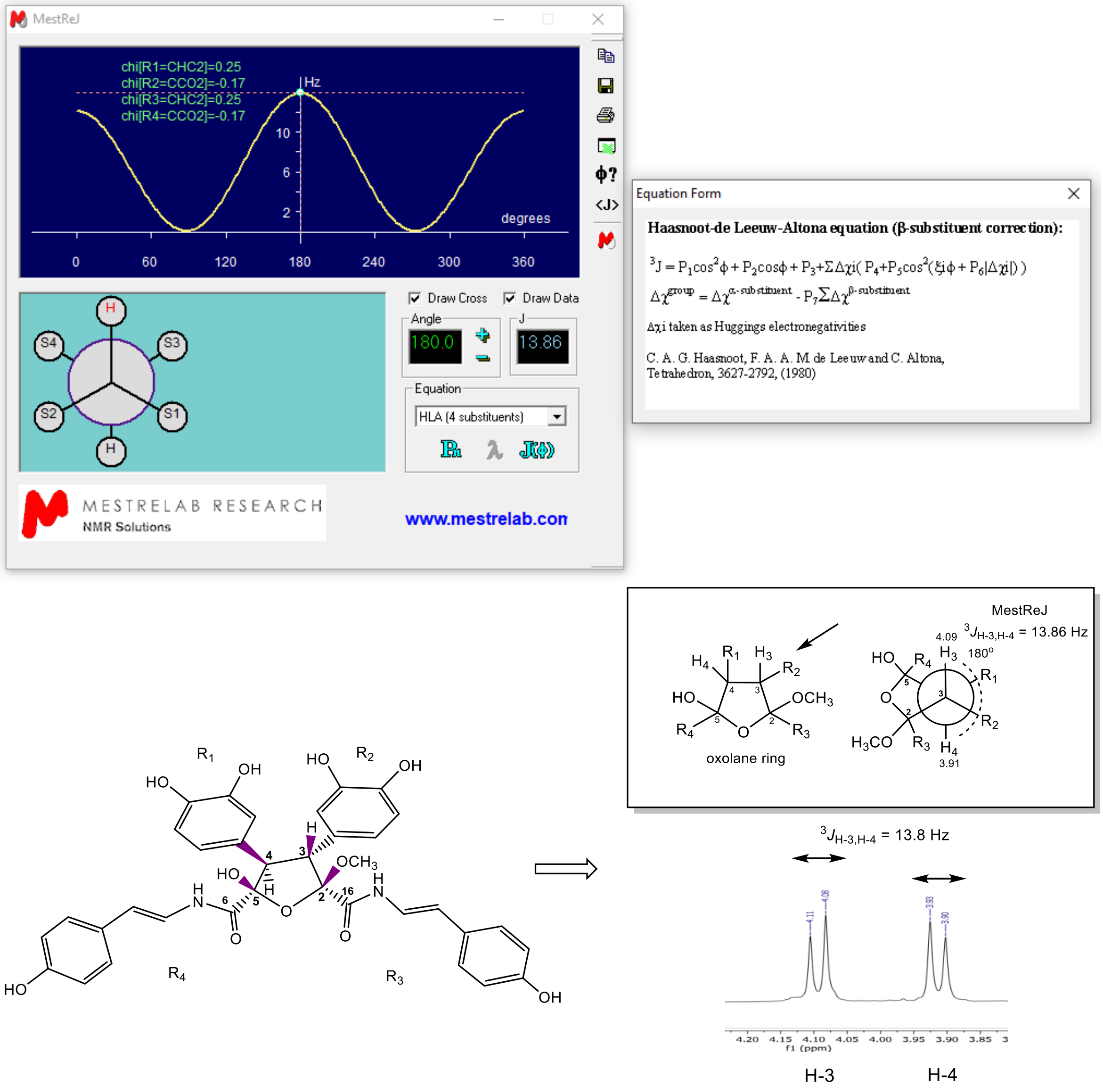

Figure S9. Calculated dihedral angle along the C-3/C-4 bond using MestReJ v1.1 for **1** in DMSO-d_6

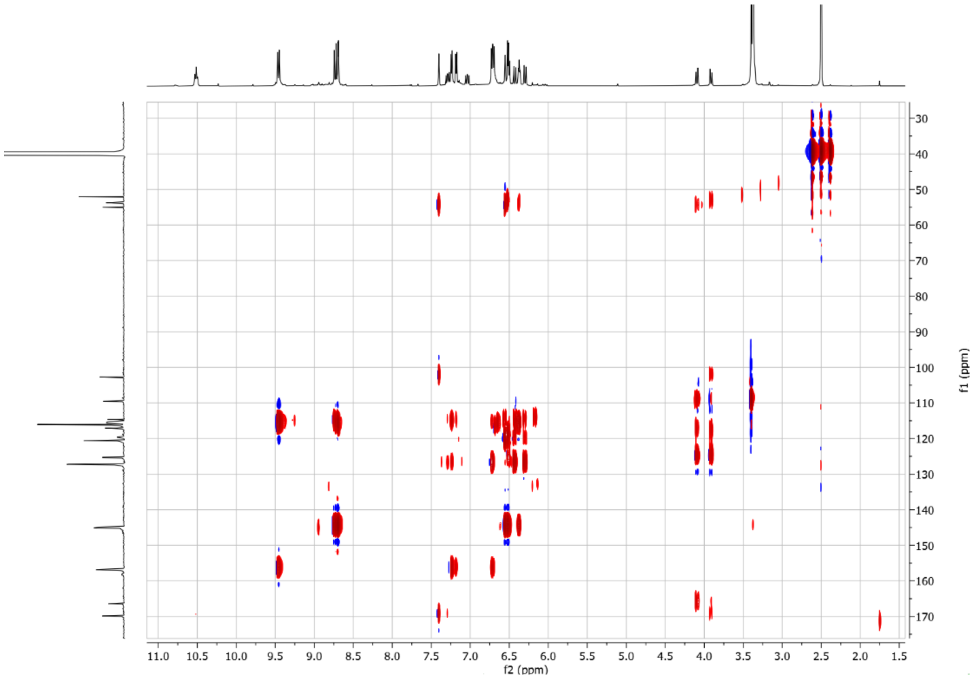

Figure S10. IP PIP-HSQMBC NMR (*cnst* 25 = 0, DMSO-d_6) spectrum of **1**

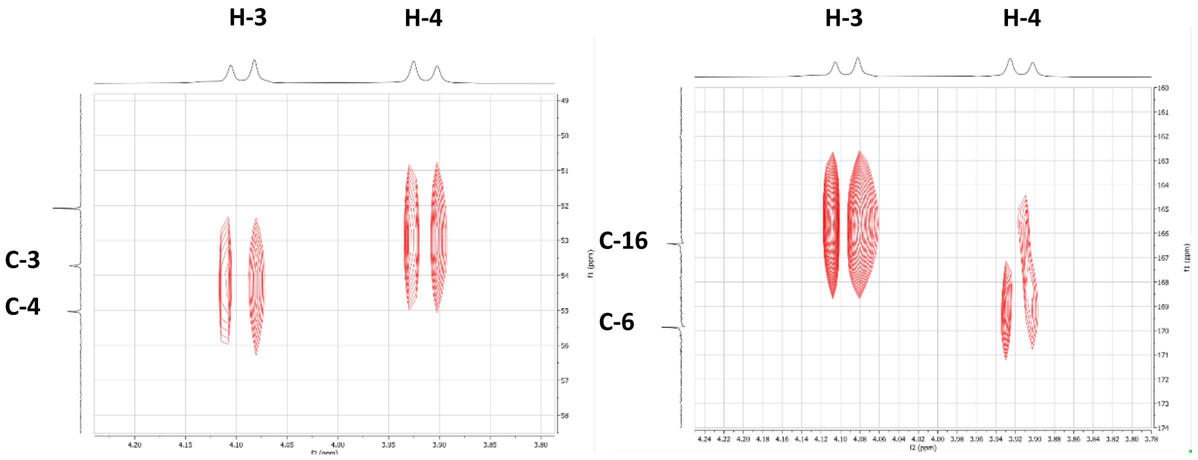

Figure S11. Expanded IP PIP-HSQMBC NMR (*cnst* 25 = 0, DMSO-d_6) spectrum of **1**

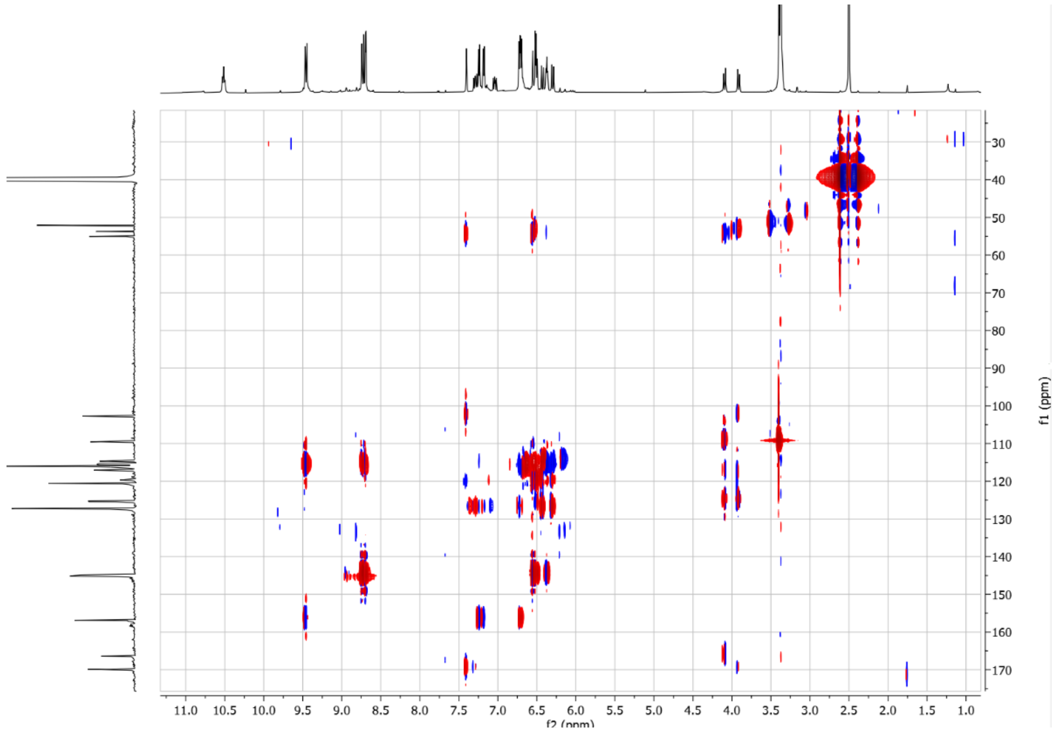

Figure S12. AP PIP-HSQMBC NMR (*cnst* 25 = 1, DMSO-d_6) spectrum of **1**

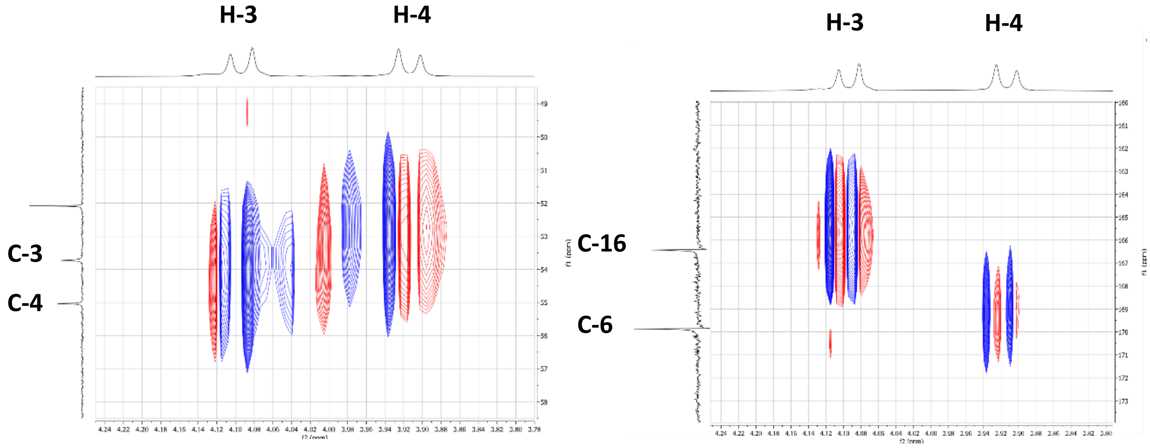

Figure S13. Expanded AP PIP-HSQMBC NMR (*cnst* 25 = 1, DMSO-d_6) spectrum of **1**

S14. IPAP: addition/subtraction procedure using "adsu" in TopSpin 4.0.7

referred from Magn. Reson. Chem. **2020**, *58*, 363–375.

Once IP dataset and AP dataset have been acquired, follow the steps below:

First thing we have to do is to create two new subspectra: copy IP experiment (let's say expno 80) into two new data set by typing "wrpa 1080" and then "wrpa 1081".

> *In 1080 we will add the AP dataset (expno81), so that 1080 will eventually be IP+AP.*
> *In 1081 we will subtract the AP dataset (expno81), so that 1081 will eventually be IP-AP.*

1. Open 1080 dataset and then type "adsu" in the command line.

2. Click on "Define 2nd dataset" in the top panel
   In the pop-up window, we type "81" (which is the AP data we want to add) in the expno box, and we type "1" in the procno box.
   Click OK.

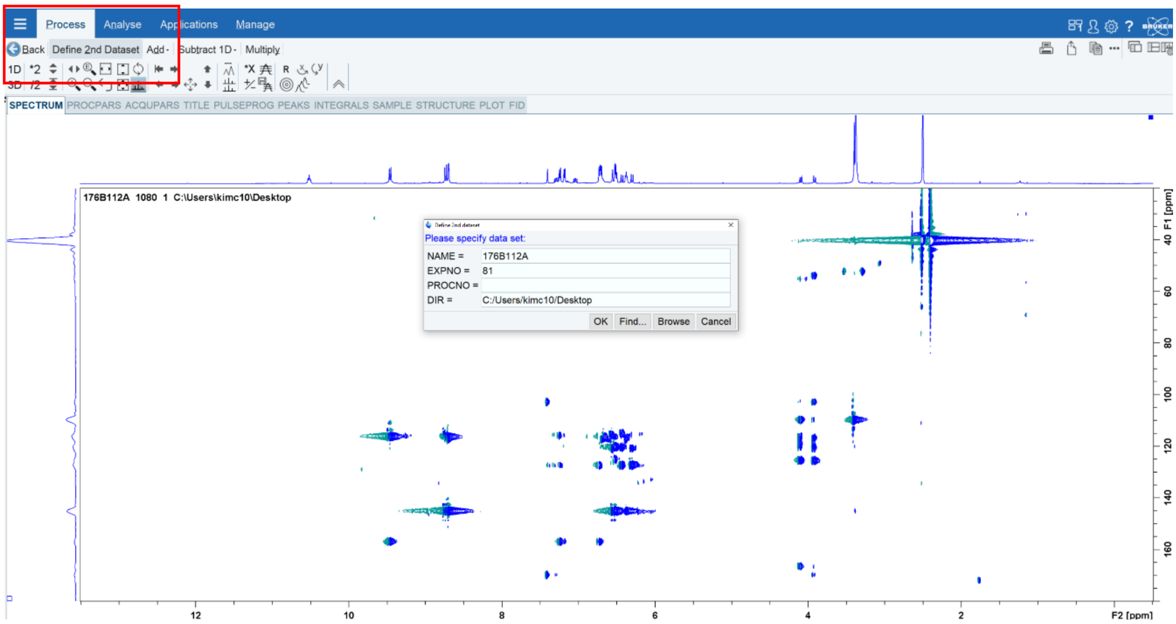

3. We then click on "Add" in the top panel. <u>Make sure you click on the arrow section of the icon (indicated by the black arrow)</u>

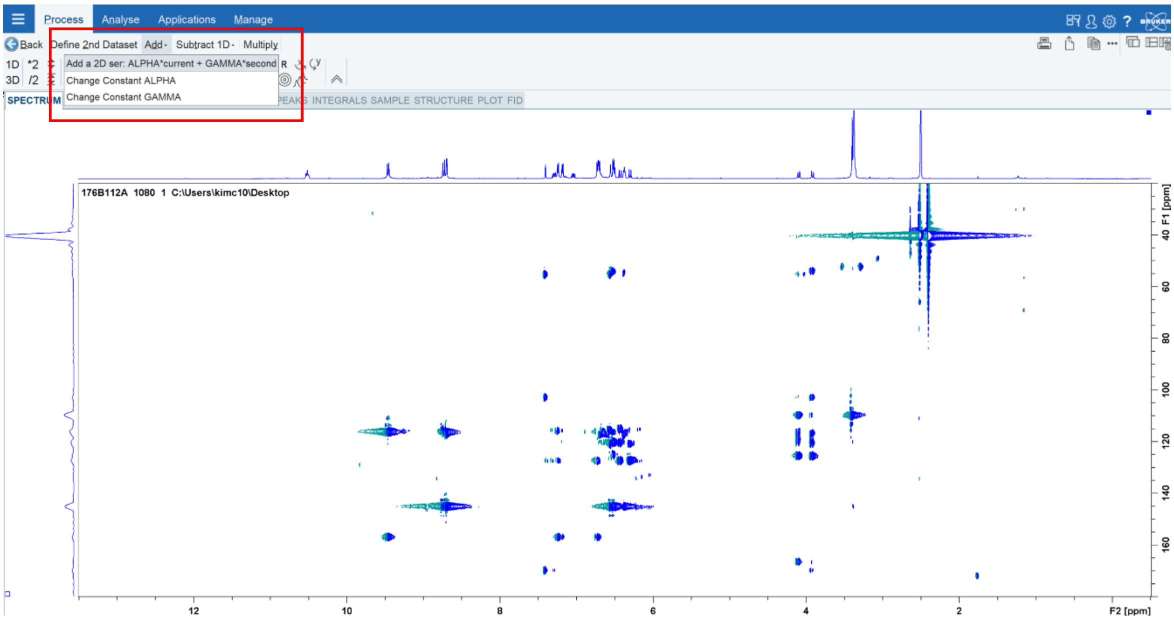

The equation is ALPHA*current (dataset) + GAMMA*second (dataset).

Since we are now <u>adding</u> the second dataset to get the IP+AP subspectra, we set ALPHA and GAMMA both to be 1 by clicking “change constant ALPHA” and “change constant GAMMA”, respectively.

4. When ALPHA and GAMMA have been set, we then click on “Add a 2D ser” option. <u>Again, make sure you click on the arrow section of the icon (indicated by the black arrow)</u>

   TopSpin will ask if we are sure we want to add, we click OK.

5. We then process the data typing “xfb”.

6. We now open 1081 dataset and repeat steps 2-5 with the only exception that GAMMA must be set to -1 in this case as we want to do the <u>subtraction</u> step here to get the IP-AP subspectra.

We now have both subspectra (IP+AP, and IP-AP) ready to be analyzed.

After **extracting 1D slices** at the carbon frequencies of interest from the IP+AP dataset (1080) and IP-AP dataset (1081), we can overlap them to measure with high accuracy the long-range proton-carbon coupling constant just by looking at their relative displacement.

Extracting 1D slices at the carbon frequencies

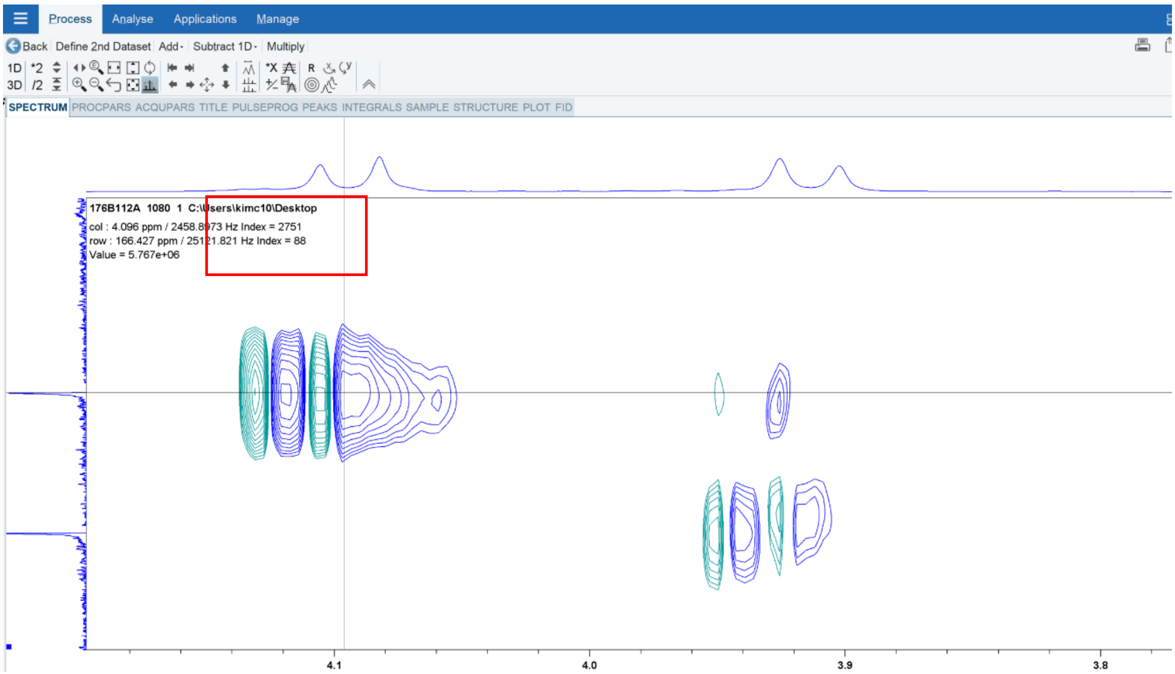

We need to write down for the index value of interesting signals. Then, we click on “Advanced” in the top panel and Extract Rows/Columns (slice).

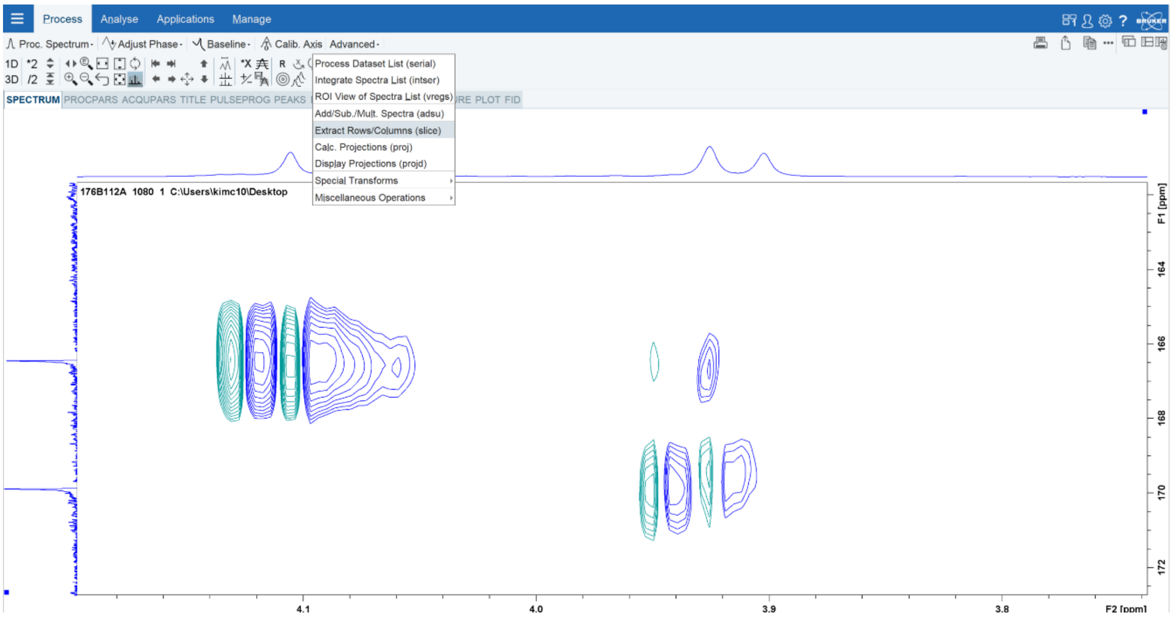

Lastly, we put the index value into the Row/column and designated 2 as a PROCNO number.

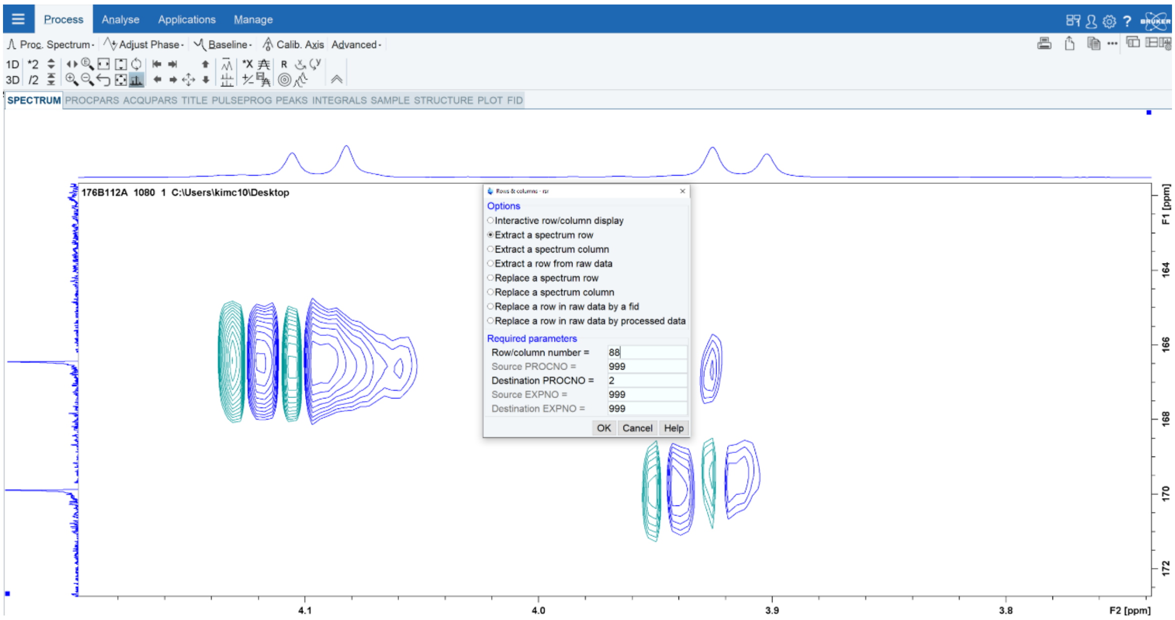

<u>Peak overlapping</u>

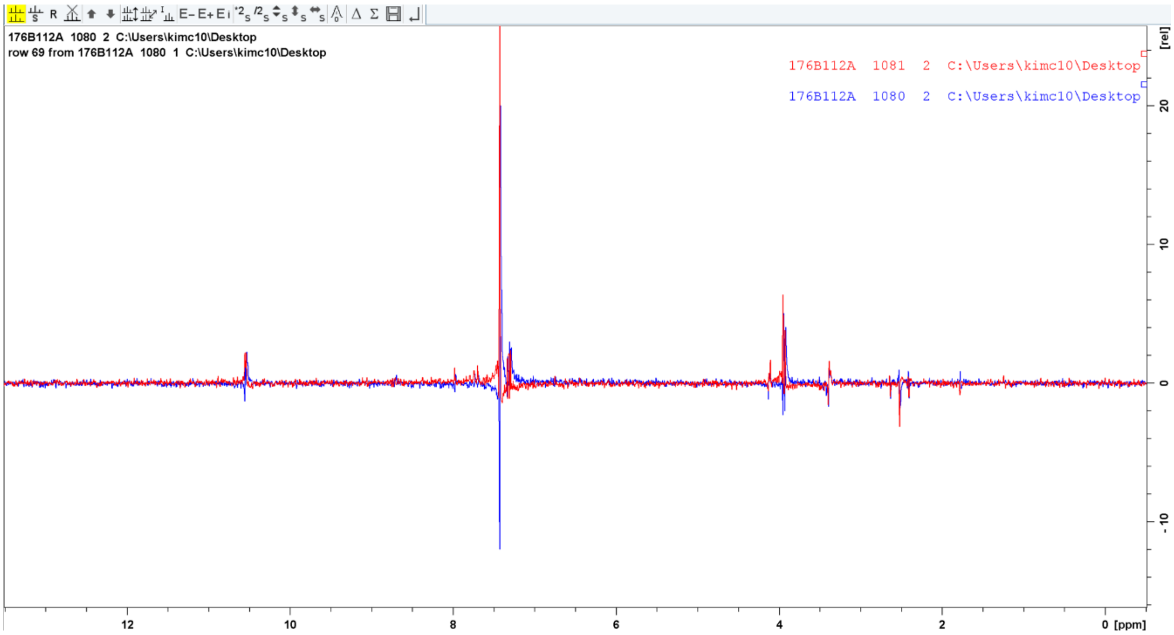

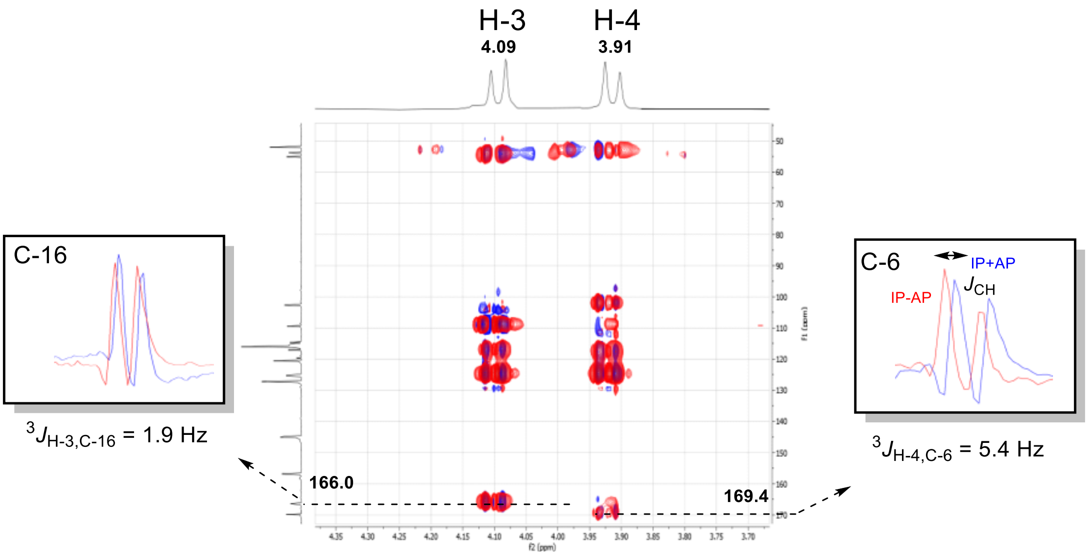

2D PIP-HSQMBC-IPAP

-extracting 1D slices at the carbon frequencies

-relative sign information cannot be extracted from the PIP-HSQMBC experiment.

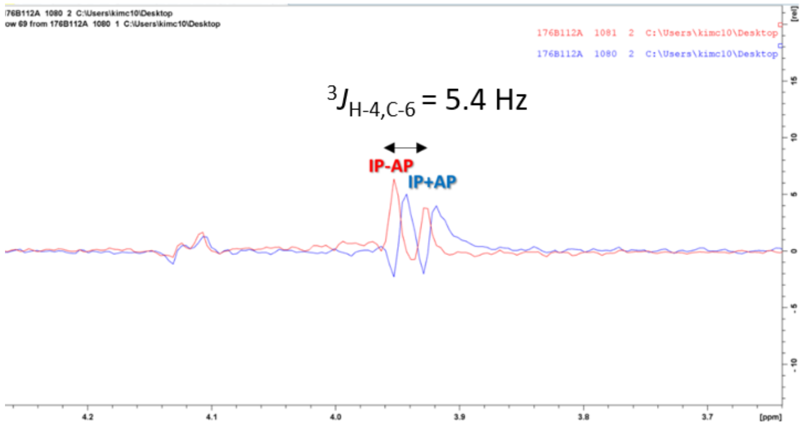

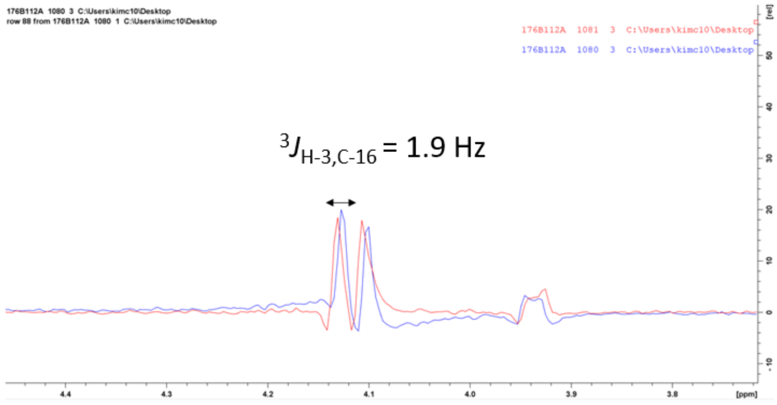

Figure S15. Extracted 1D carbon slices (DMSO-d_6) from spectra of **1**

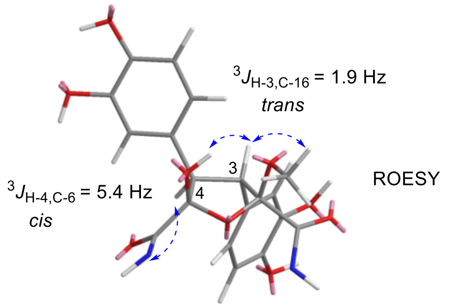

J measured	value (Hz)	relative orientation
1) $^{3}J_{H-3,C-16}$	1.9	C-H *trans*
2) $^{3}J_{H-4,C-6}$	5.4	C-H *cis*

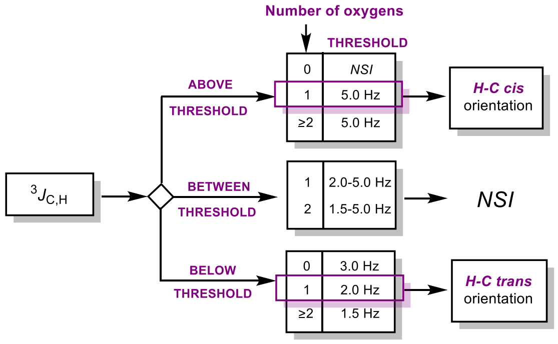

(NSI: no stereochemical information can be extracted).

Chem. Eur. J. **2011**, *17*, 6338 - 6347

Figure S16. Flow chart to use $^{3}J_{C,H}$ values for configurational analysis of five-membered rings.

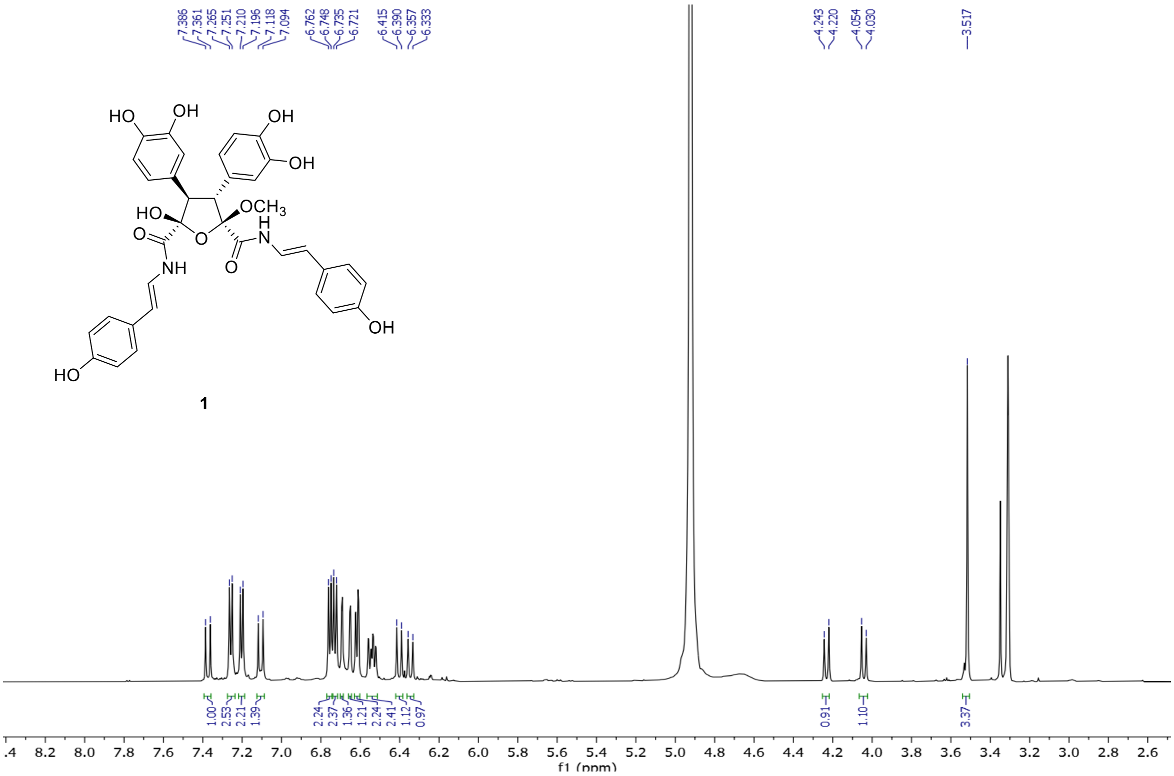

Figure S17. ^{1}H NMR (600 MHz, MeOH-d_4) spectrum of **1**

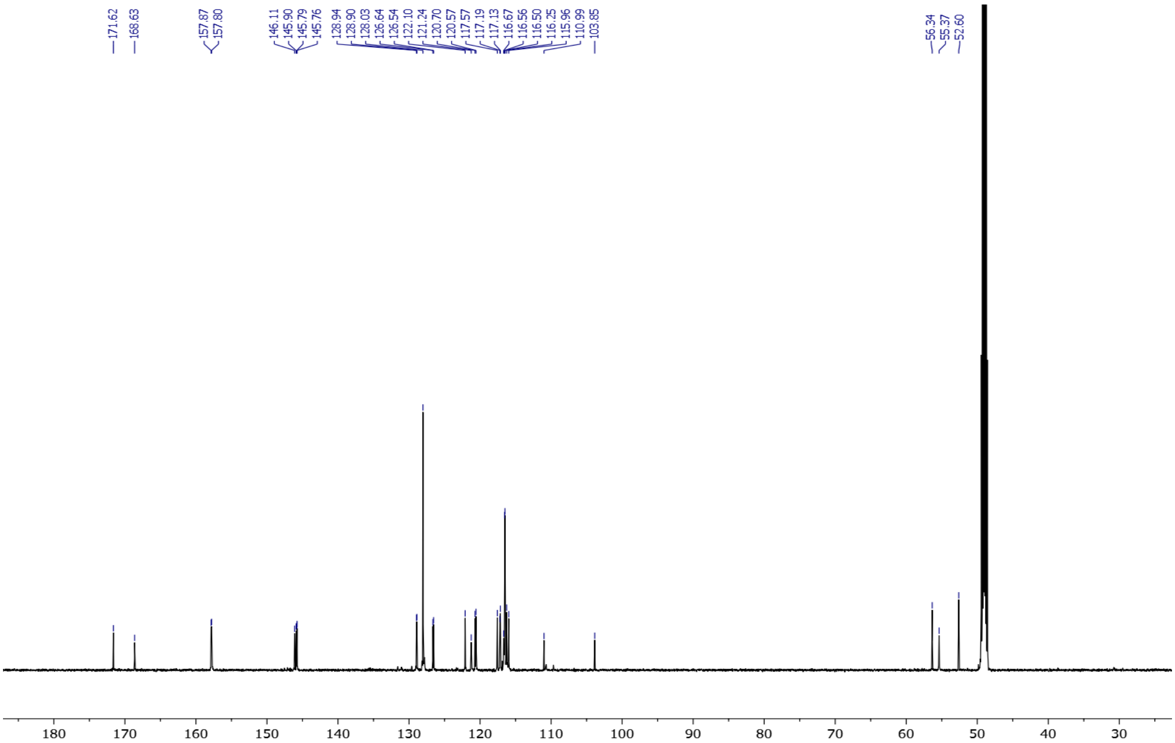

Figure S18. ^{13}C NMR (150 MHz, MeOH-d_4) spectrum of **1**

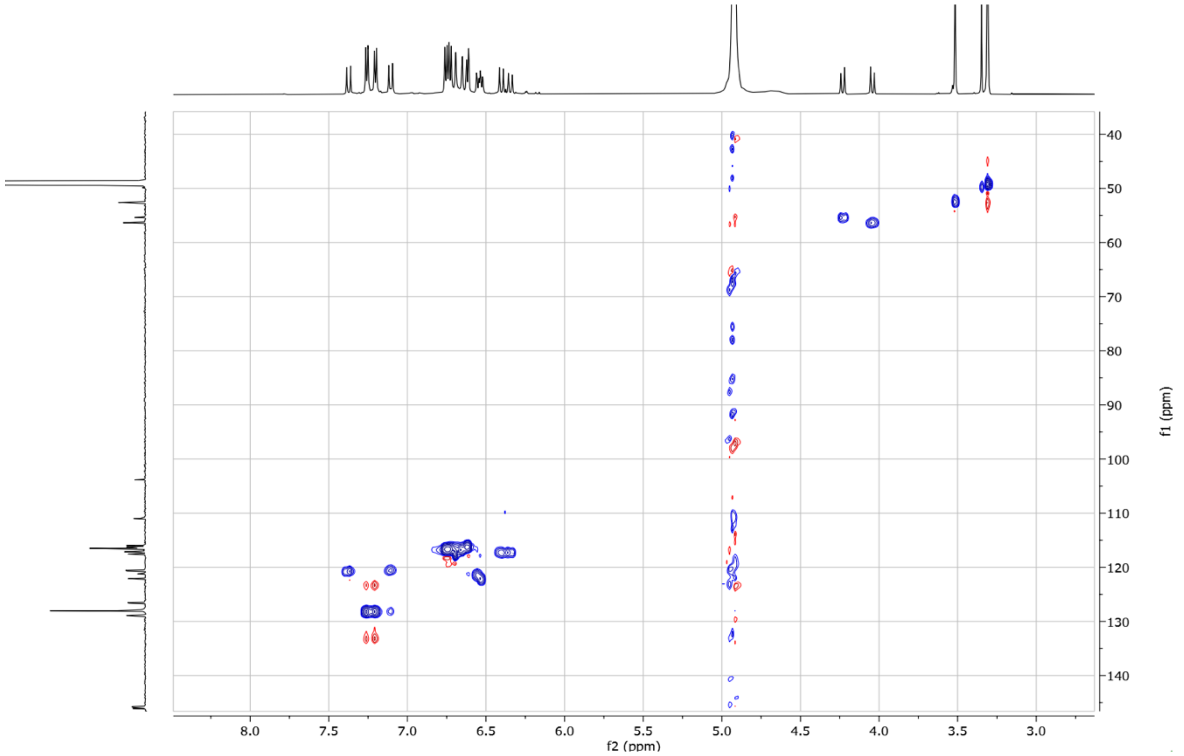

Figure S19. Edited HSQC NMR (MeOH-d_4) spectrum of **1**

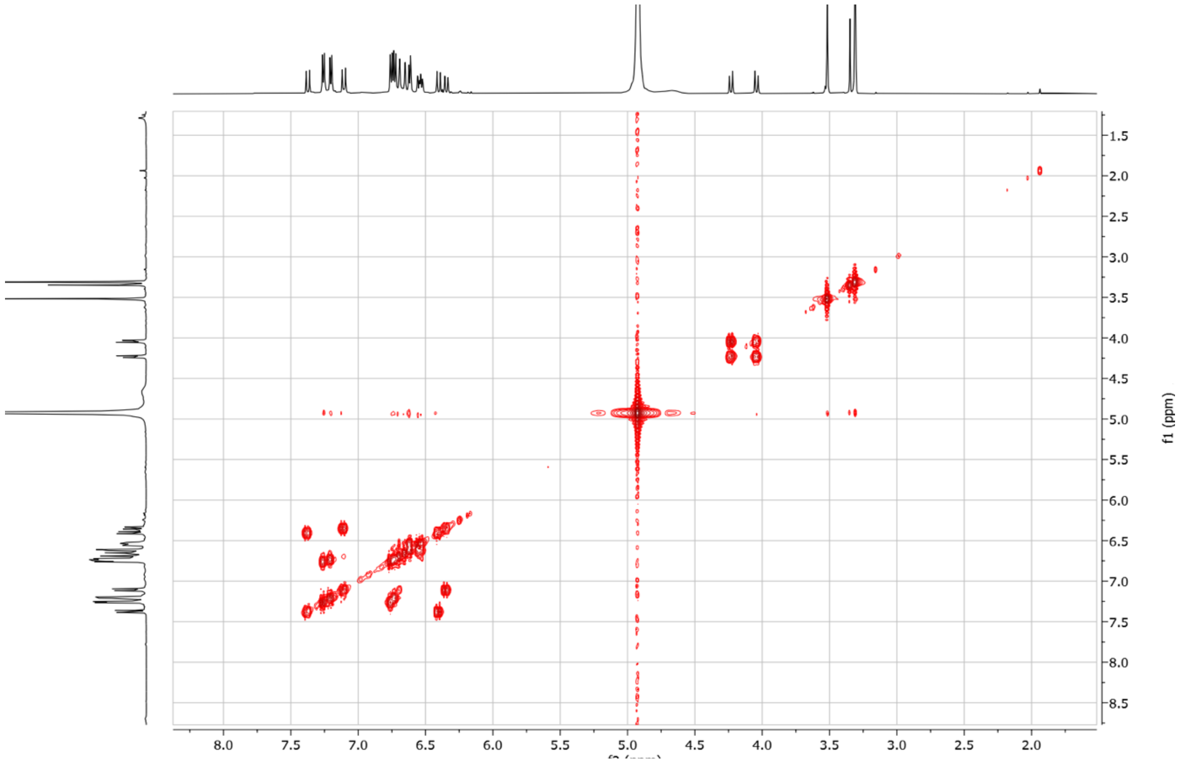

Figure S20. COSY NMR (600 MHz, MeOH-d_4) spectrum of **1**

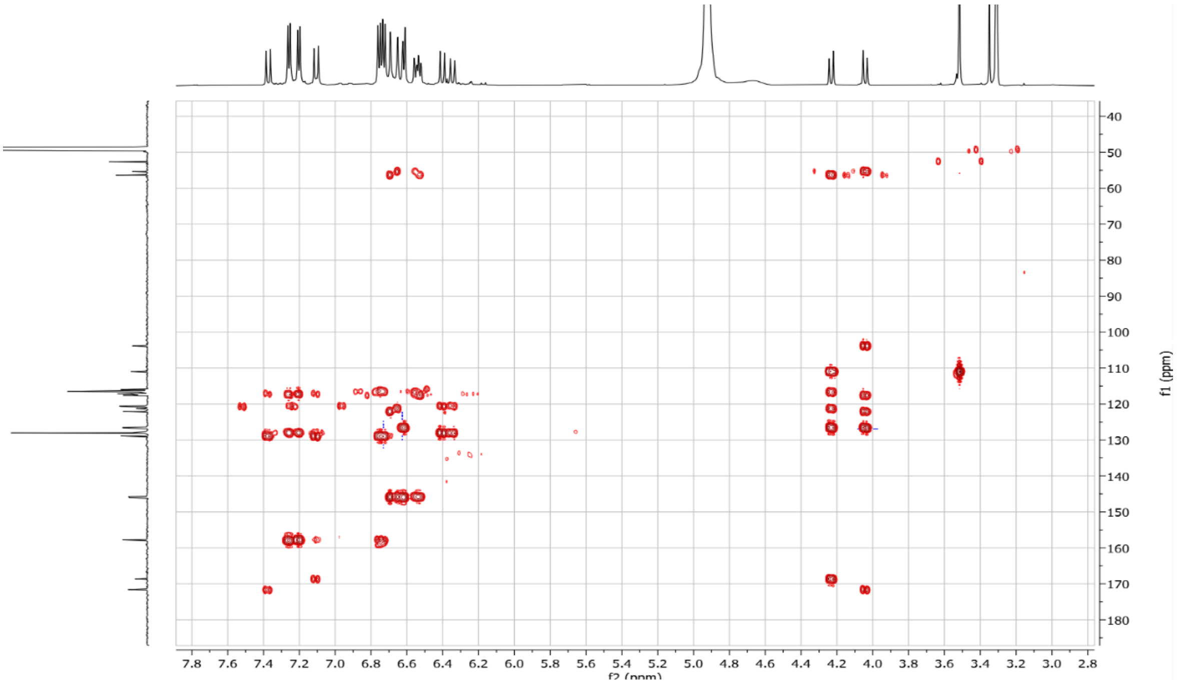

Figure S21. HMBC NMR (MeOH-d_4) spectrum of **1**

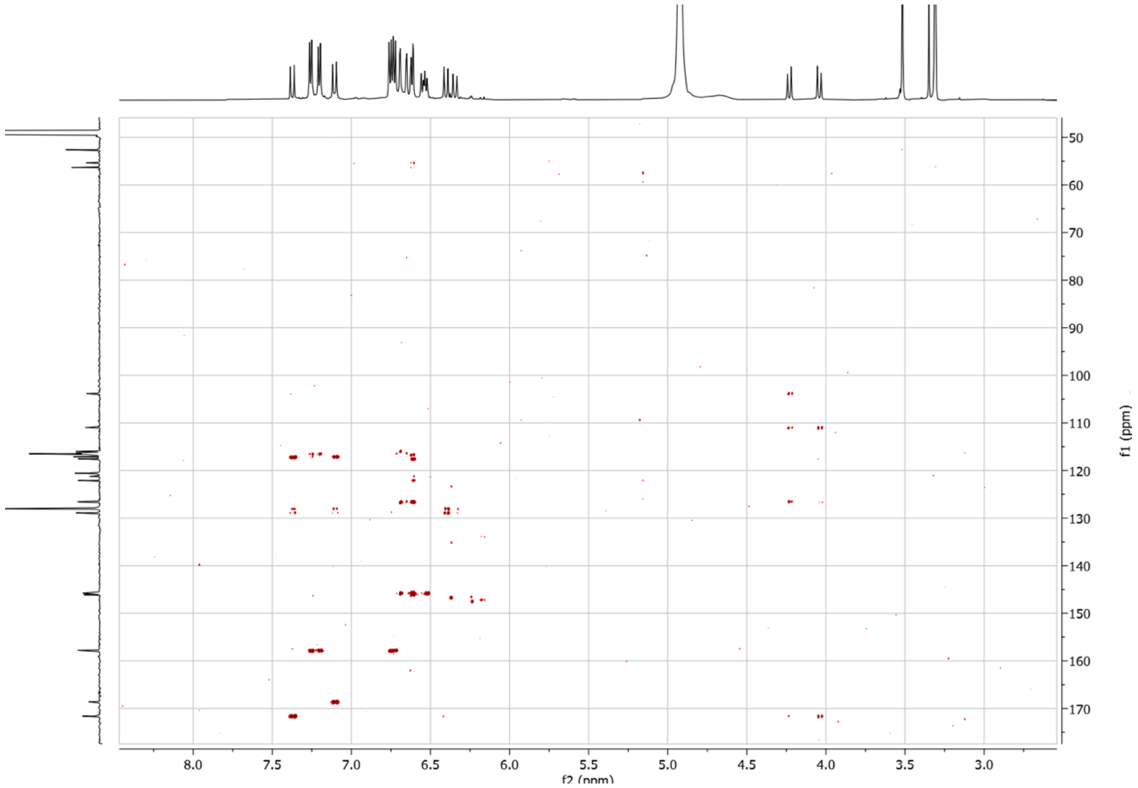

Figure S22. LR-HSQMBC NMR (MeOH-d_4) spectrum of **1**

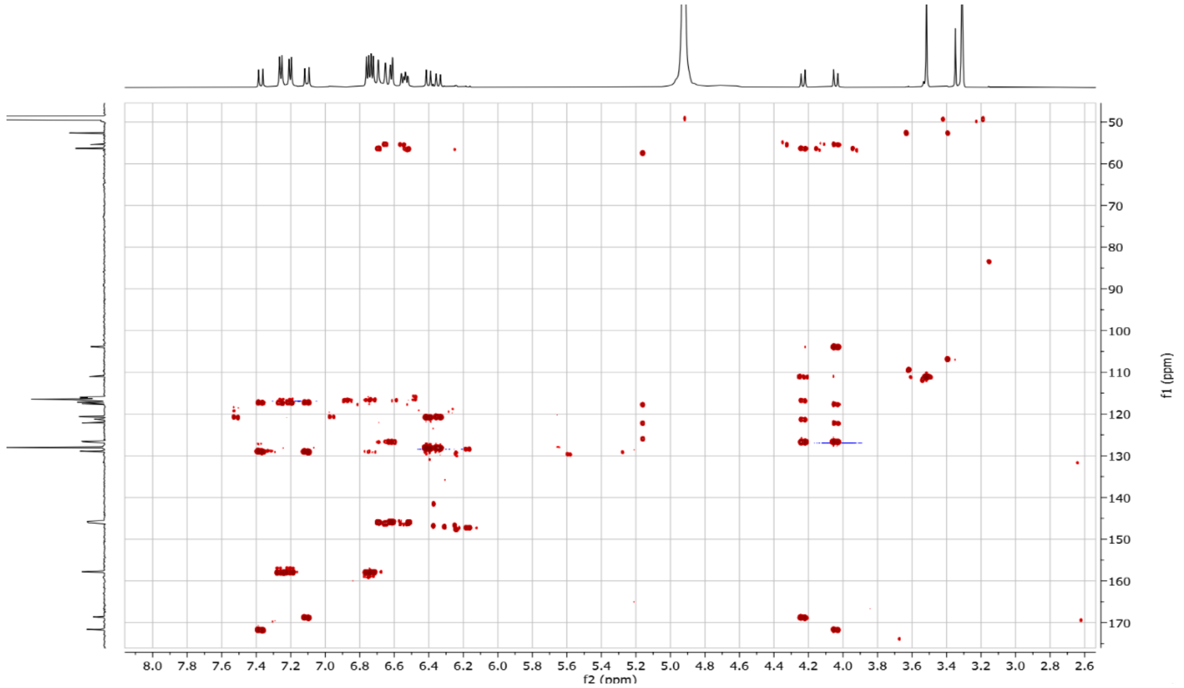

Figure S23. HMBC NMR (J_{CH} = 3.5 Hz, MeOH-d_4) spectrum of **1**

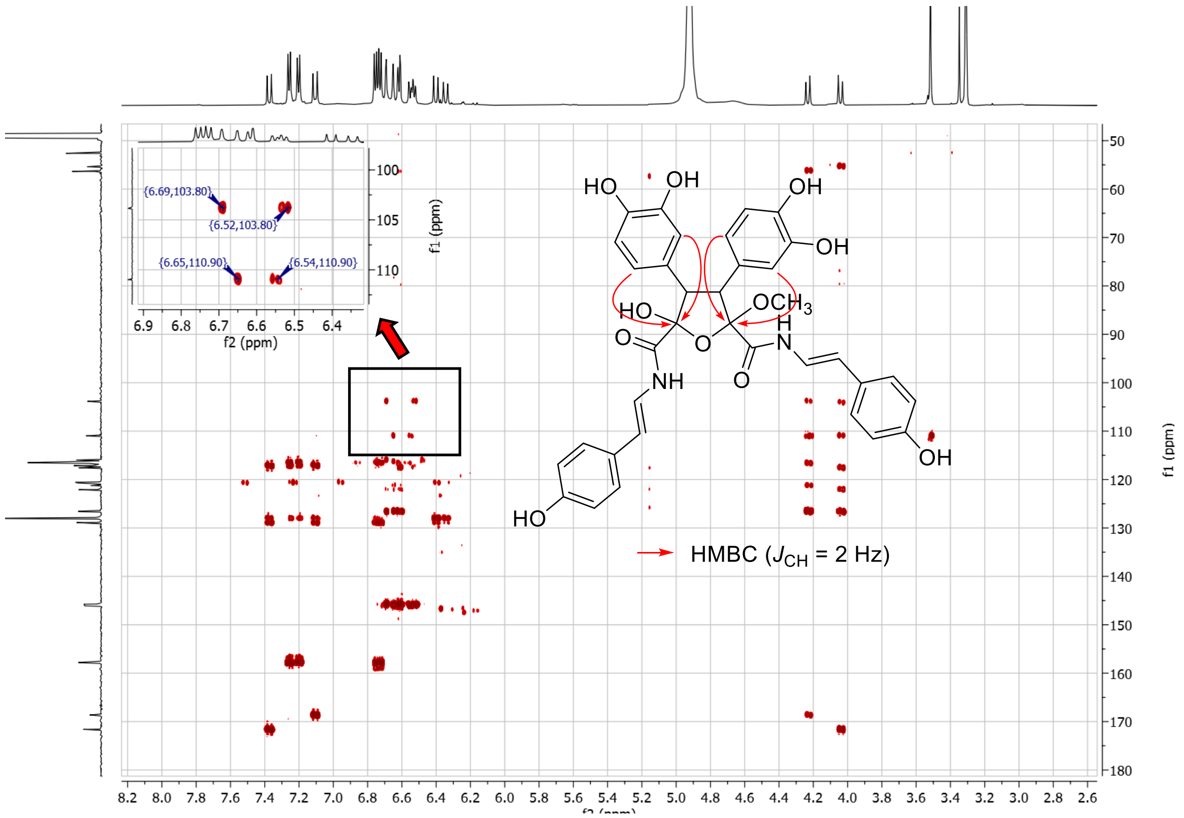

Figure S24. HMBC NMR (J_{CH} = 2.0 Hz, MeOH-d_4) spectrum of **1**

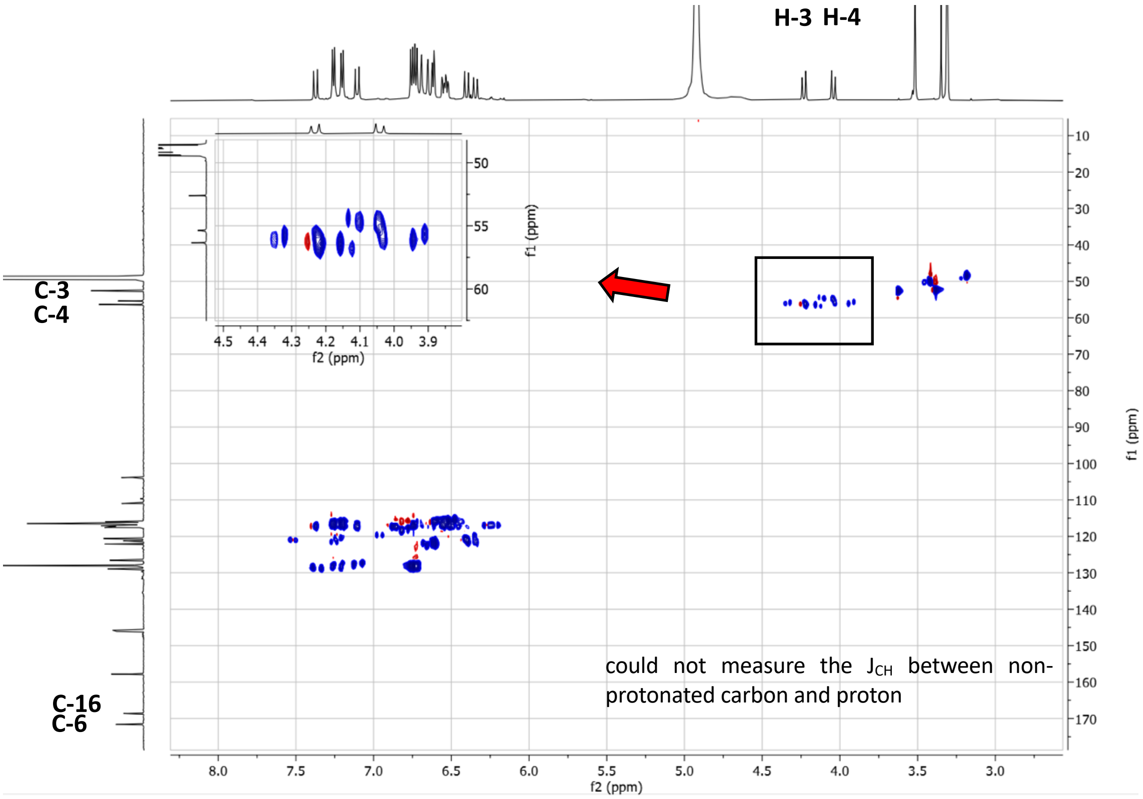

Figure S25. HSQC-HECADE NMR (MeOH-d_4) spectrum of **1**

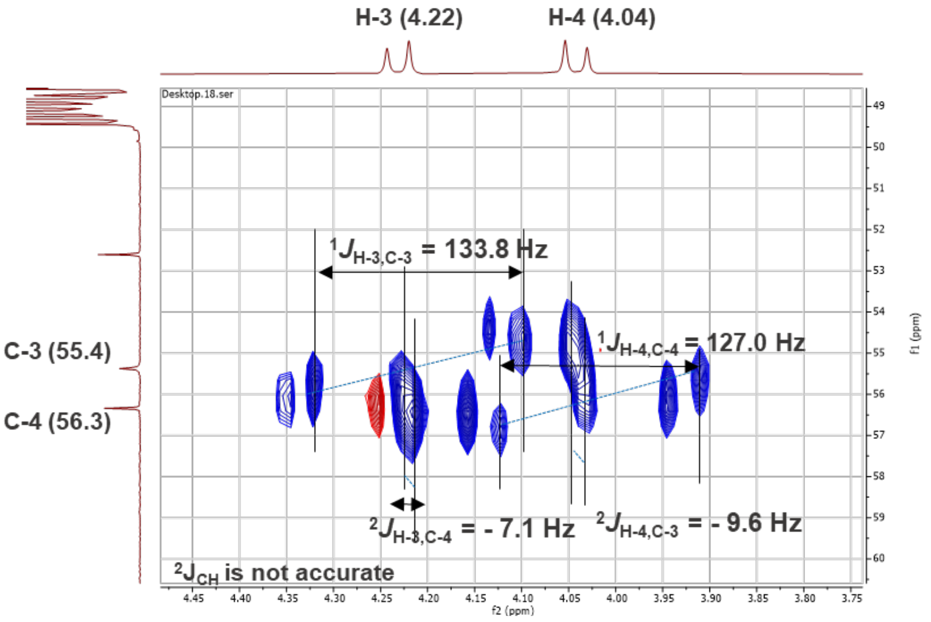

all positive couplings will be tilted as "/", while negative couplings will form a backward slash "\"

Figure S26. Expanded HSQC-HECADE NMR (MeOH-d_4) spectrum of **1**

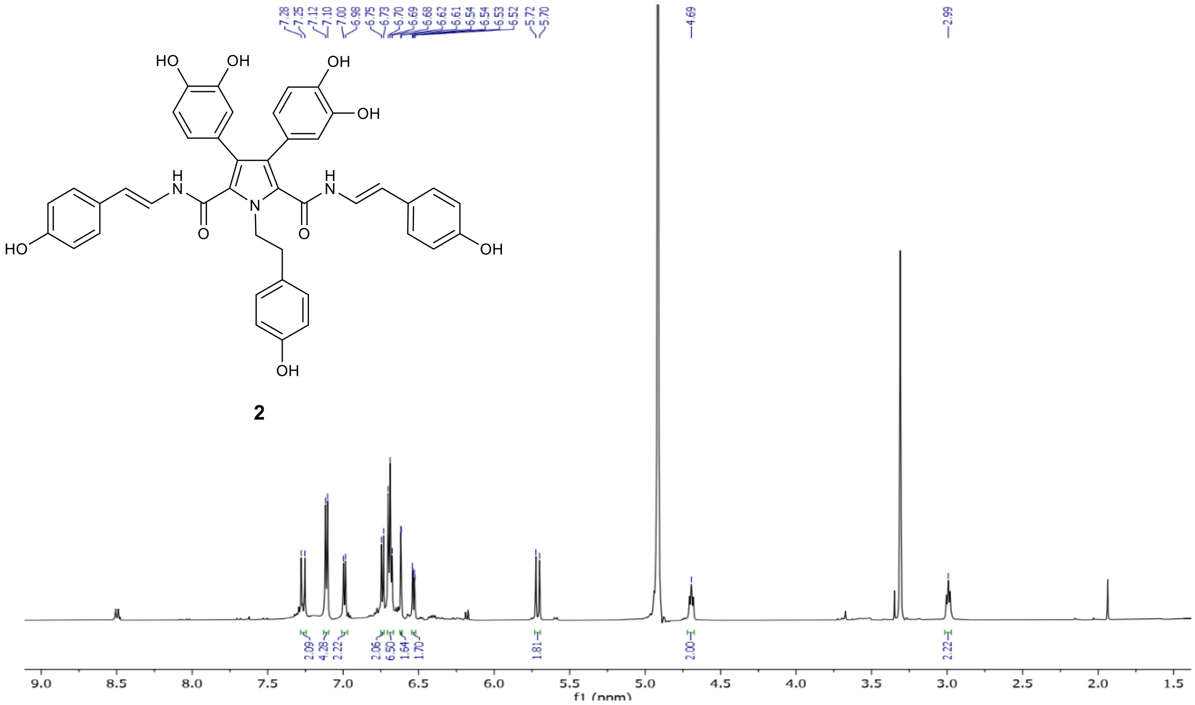

Figure S27. ^{1}H NMR (600 MHz, MeOH-d_4) spectrum of **2**

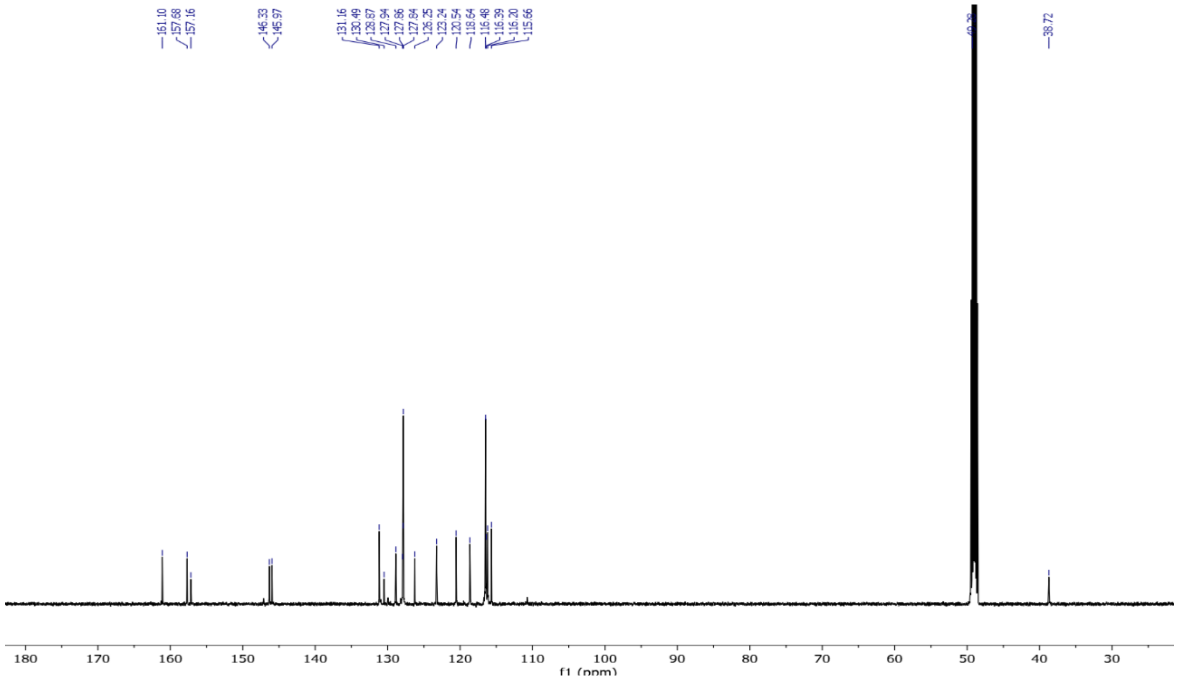

Figure S28. ^{13}C NMR (150 MHz, MeOH-d_4) spectrum of **2**

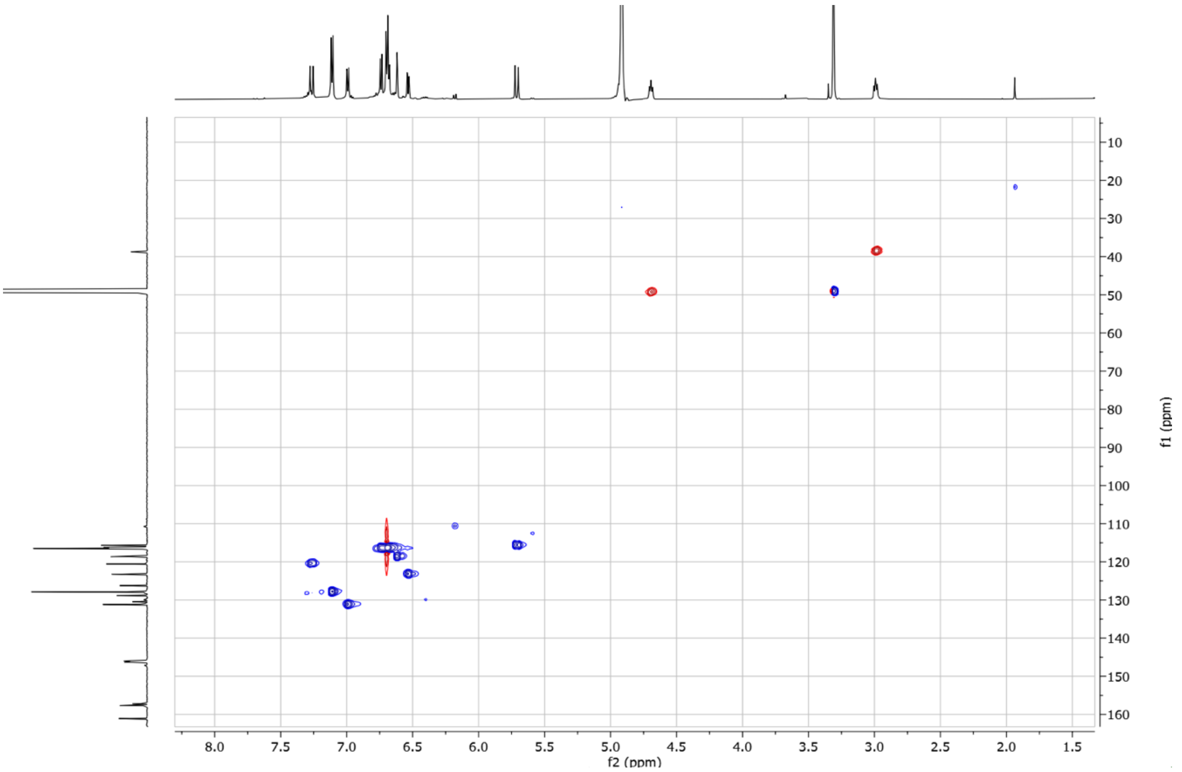

Figure S29. Edited HSQC NMR (MeOH-d_4) spectrum of **2**

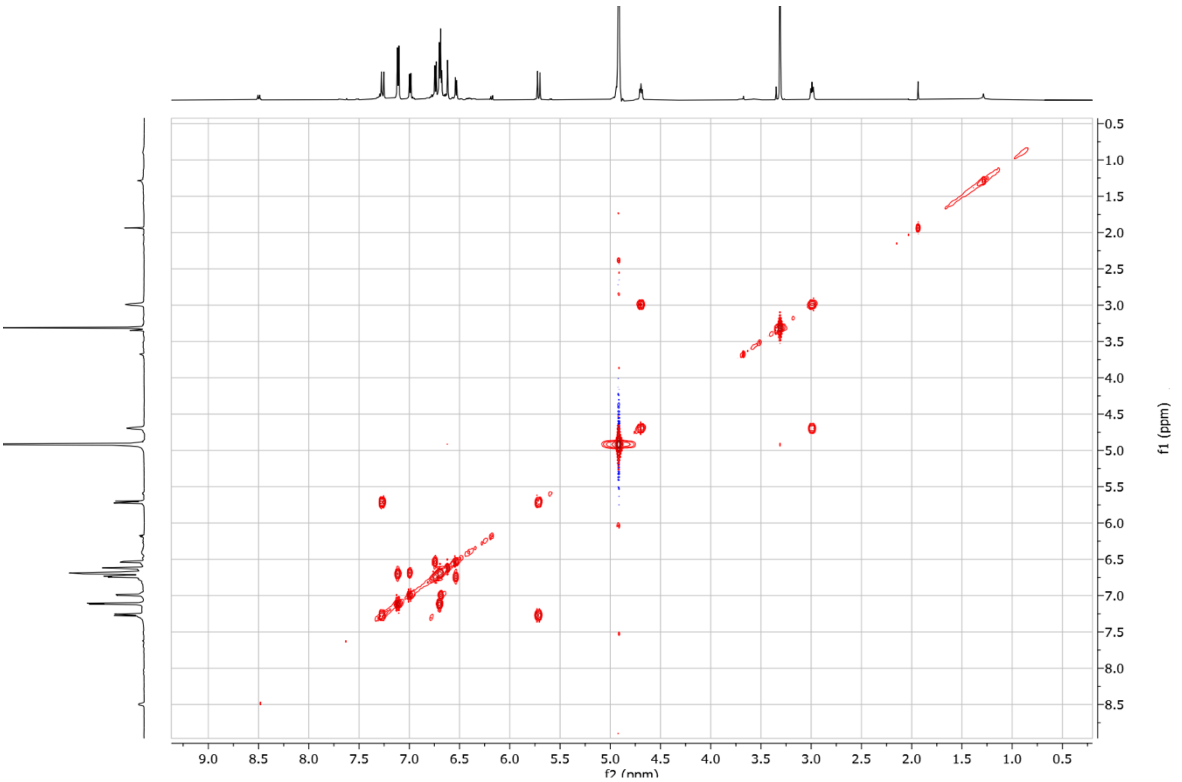

Figure S30. COSY NMR (600 MHz, MeOH-d_4) spectrum of **2**

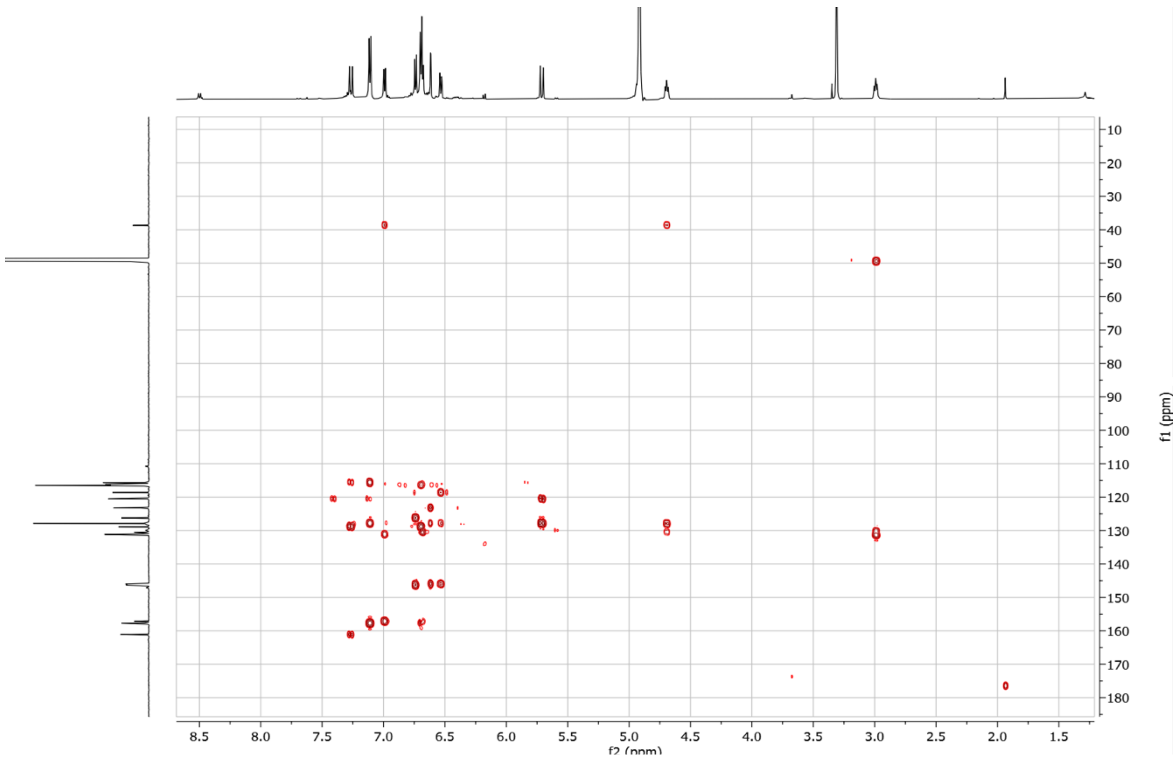

Figure S31. HMBC NMR (MeOH-d_4) spectrum of **2**

Analysis Report

Agilent | Trusted Answers

Sample Information

**Name**	176B09814_2	**Data File Path**	C:\Users\Public\Desktop\QTOF data\Data\Chang\100819\176B09814_2.d
**Sample ID**		**Acq. Time (Local)**	10/8/2019 9:04:25 AM (UTC-04:00)
**Instrument**	Instrument 1	**Method Path (Acq)**	D:\MassHunter\Methods\FIA_SM_LowFlow.m
**MS Type**	QTOF	**Version (Acq SW)**	6200 series TOF/6500 series Q-TOF B.09.00 (B9044.1 SP1)
**Inj. Vol. (ul)**	1	**IRM Status**	Some ions missed
**Position**	Vial 17	**Method Path (DA)**	
**Plate Pos.**		**Target Source Path**	
**Operator**		**Result Summary**	

Sample Spectra

+ Scan (rt: 0.360-0.807 min) **C42 H35 N3 O9**

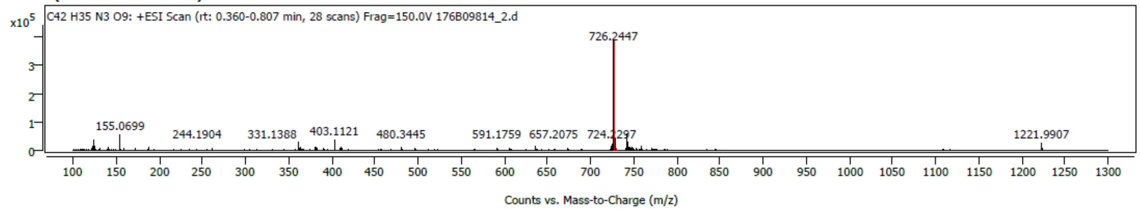

Spectrum Identification Table

Best ID Source	Name	Formula	Species	m/z	Diff (ppm)	CAS	Score	Score (Lib)	Score (DB)	Score (MFG)	Lib/DB
Yes MFG		C42 H35 N3 O9	(M+H)+	726.2447	0.05		98.47			98.47	

Figure S32. ESI- HRMS data of **2**

IR Spectrum Report

Instrument type	Alpha II
Accessory	ATR platinum Diamond 1 Refl #24C8F5322D
Spectrum file name	176B014_2-1.0
Spectrum file path	T:\Data\MEAS\Chang
Measurement date and time	15/10/2019 23:34:56 (GMT-7)
Sample name	176B014_2-1
Sample form	Instrument type and / or accessory

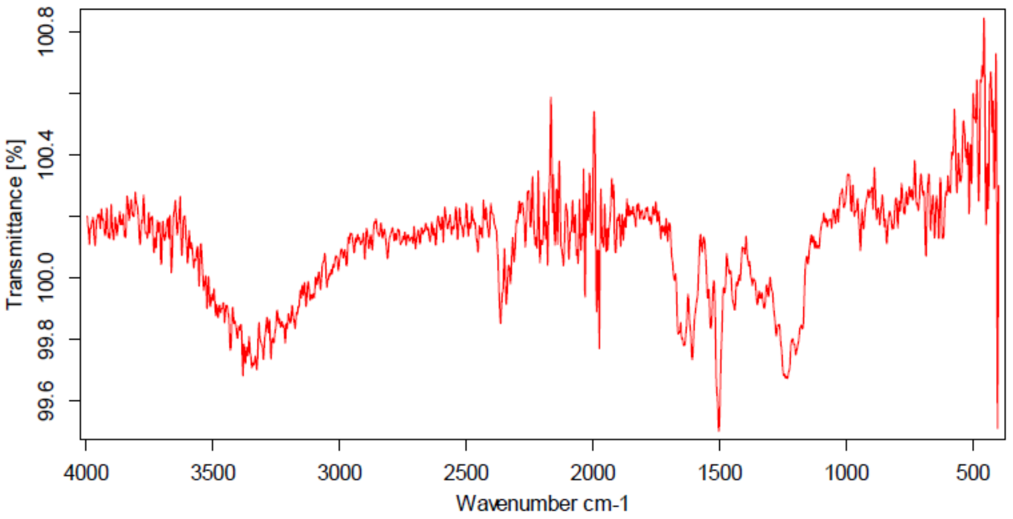

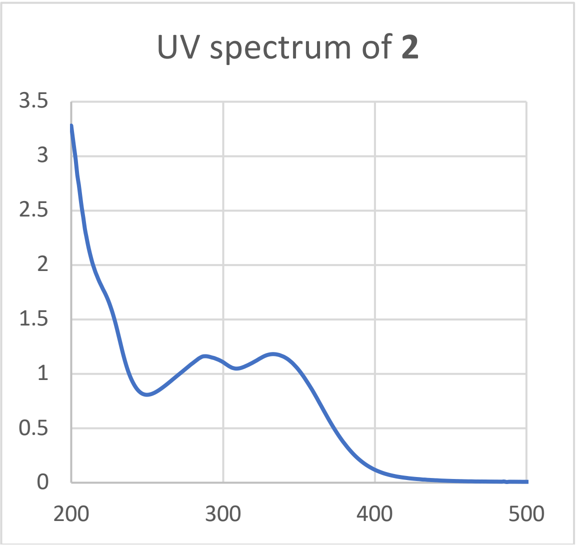

Figure S33. IR & UV spectra of **2**

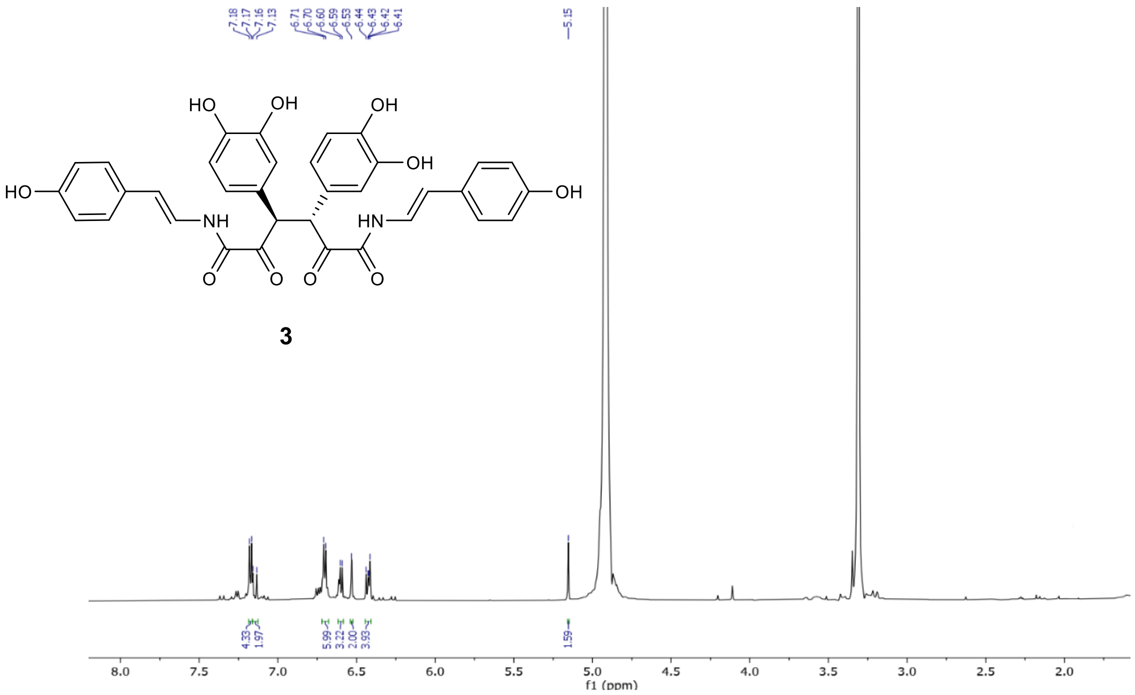

Figure S34. ^{1}H NMR (600 MHz, MeOH-d_4) spectrum of **3**

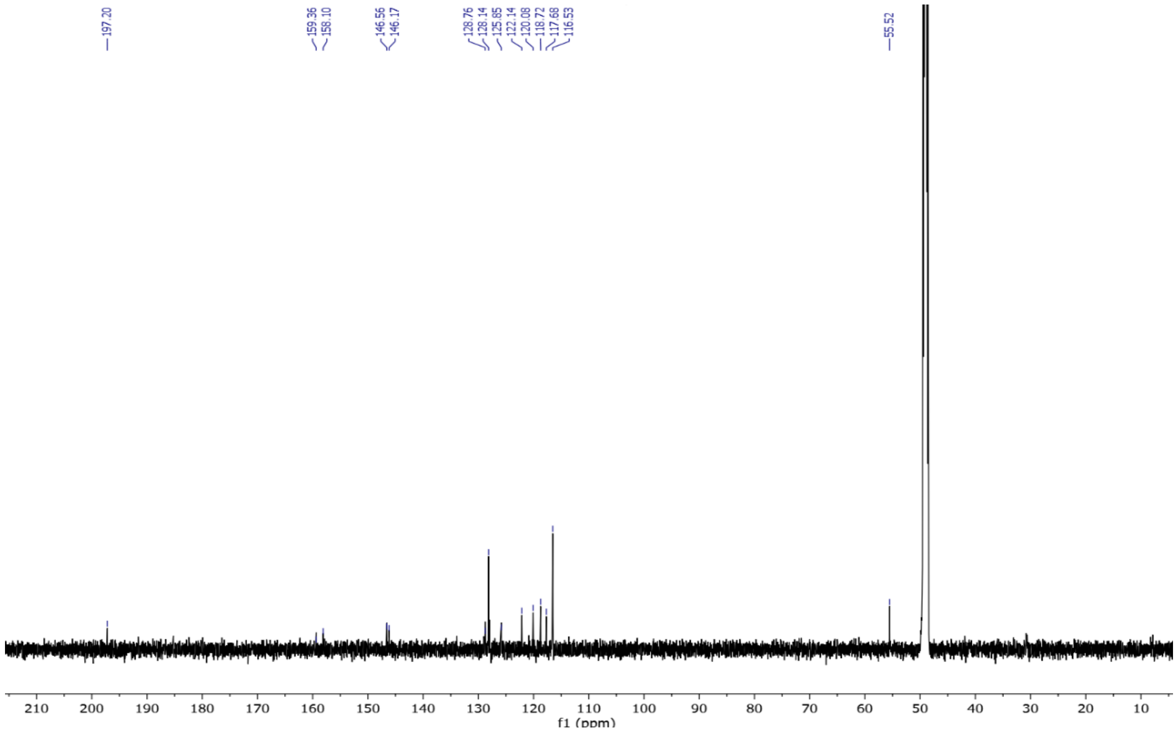

Figure S35. ^{13}C NMR (150 MHz, MeOH-d_4) spectrum of **3**

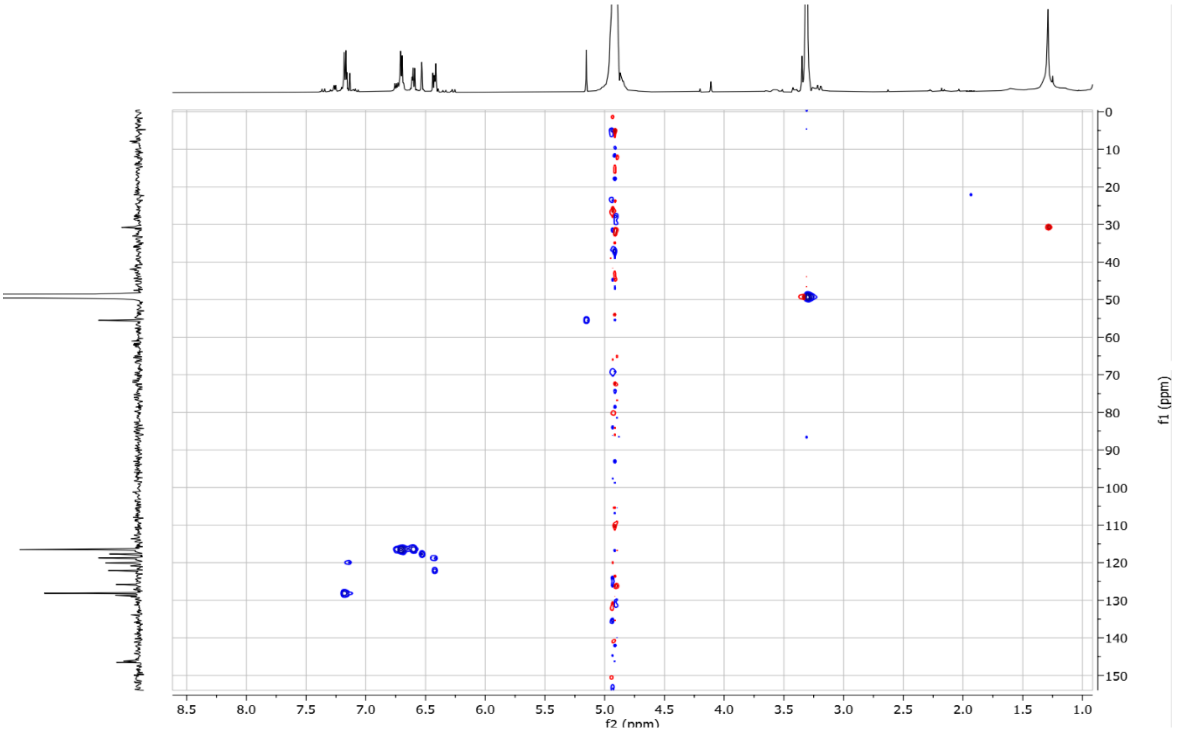

Figure S36. Edited HSQC NMR (MeOH-d_4) spectrum of **3**

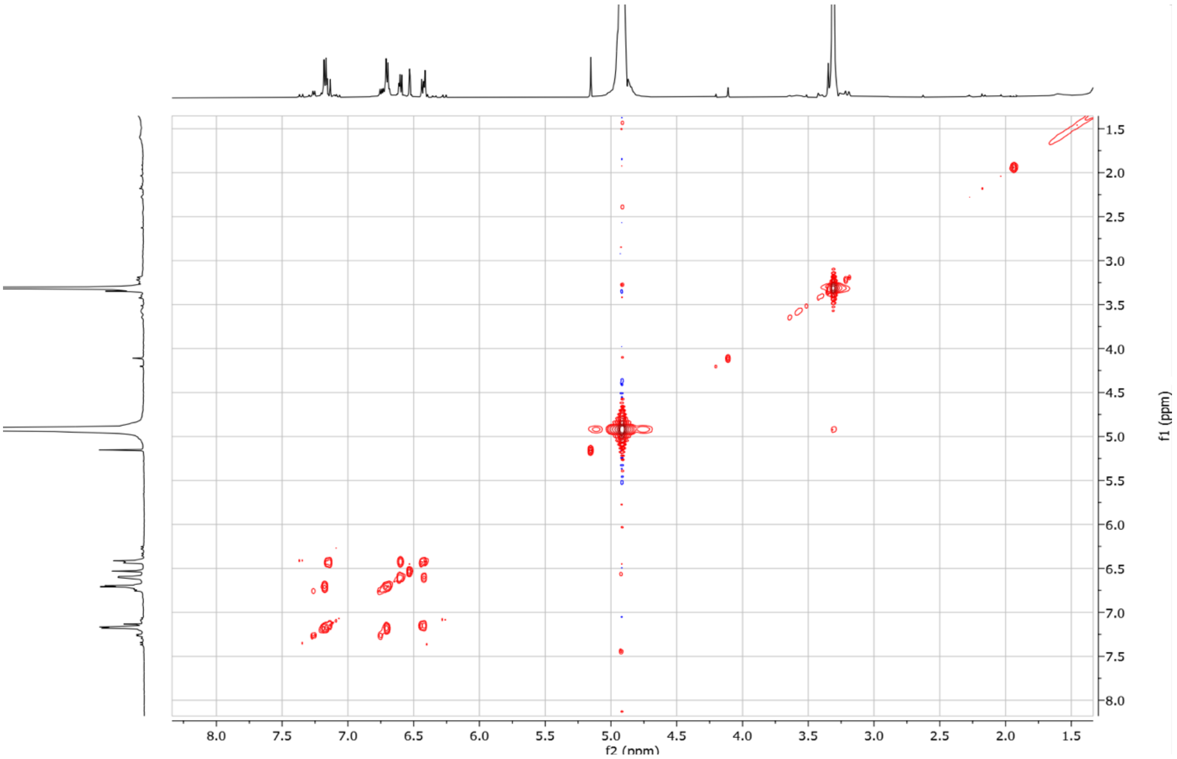

Figure S37. COSY NMR (600 MHz, MeOH-d_4) spectrum of **3**

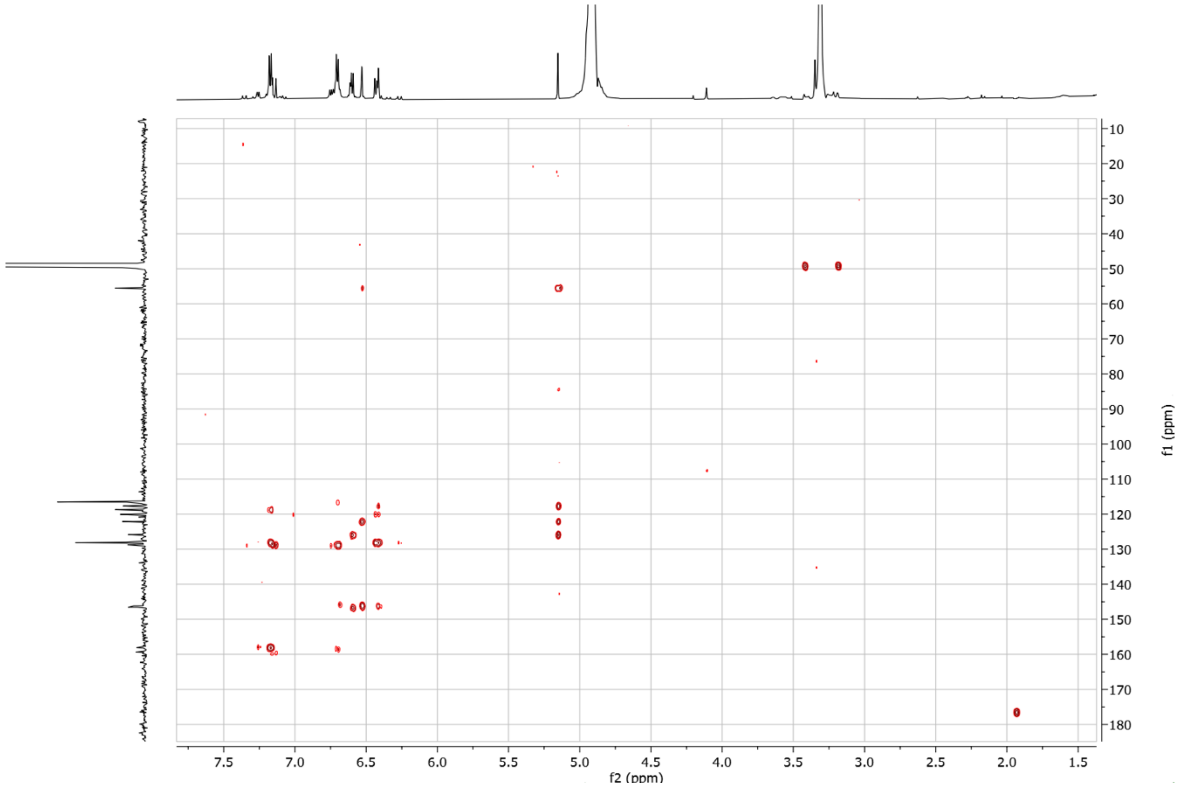

Figure S38. HMBC NMR (MeOH-d_4) spectrum of **3**

Analysis Report

Agilent | Trusted Answers

Sample Information

**Name**	176B103_8	**Data File Path**	C:\Users\Public\Desktop\QTOF data\Data\Chang\100819\176B103_8.d
**Sample ID**		**Acq. Time (Local)**	10/8/2019 9:15:21 AM (UTC-04:00)
**Instrument**	Instrument 1	**Method Path (Acq)**	D:\MassHunter\Methods\FIA_SM_LowFlow.m
**MS Type**	QTOF	**Version (Acq SW)**	6200 series TOF/6500 series Q-TOF B.09.00 (B9044.1 SP1)
**Inj. Vol. (ul)**	1	**IRM Status**	Some ions missed
**Position**	Vial 19	**Method Path (DA)**	
**Plate Pos.**		**Target Source Path**	
**Operator**		**Result Summary**	

Sample Spectra

+ Scan (rt: 0.393-0.791 min) **C34 H28 N2 O10**

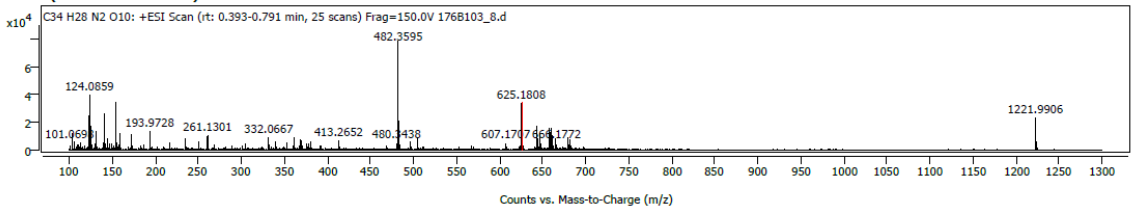

Spectrum Identification Table

Best ID Source	Name	Formula	Species	m/z	Diff (ppm)	CAS	Score	Score (Lib)	Score (DB)	Score (MFG)	Lib/DB
Yes MFG		C34 H28 N2 O10	(M+H)+	625.1808	-1.39		98.15			98.15	

Figure S39. ESI- HRMS data of **3**

IR Spectrum Report

Instrument type	Alpha II
Accessory	ATR platinum Diamond 1 Refl #24C8F5322D
Spectrum file name	176B103_8-1.3
Spectrum file path	T:\Data\MEAS\Chang
Measurement date and time	15/10/2019 23:48:57 (GMT-7)
Sample name	176B103_8-1
Sample form	Instrument type and / or accessory

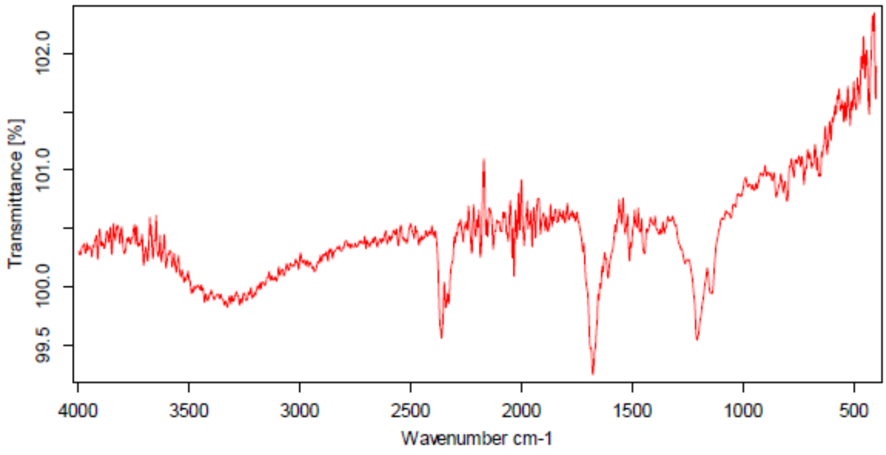

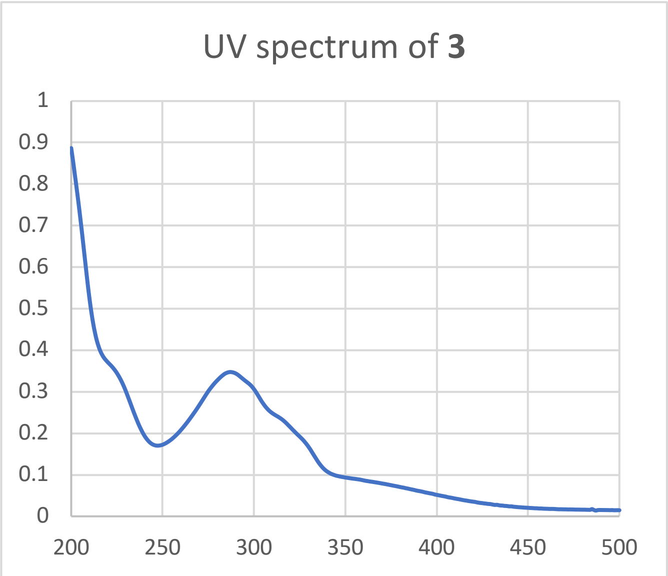

Figure S40. IR & UV spectra of **3**

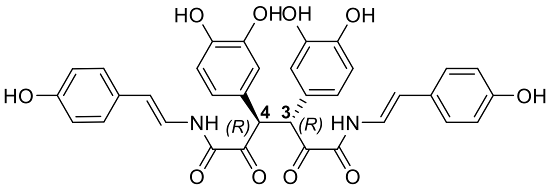

3

$[\alpha]_D$ = +86.7

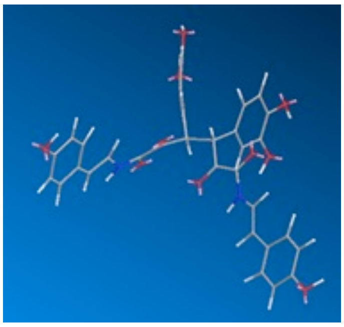

Energy minimization model using Chem 3D

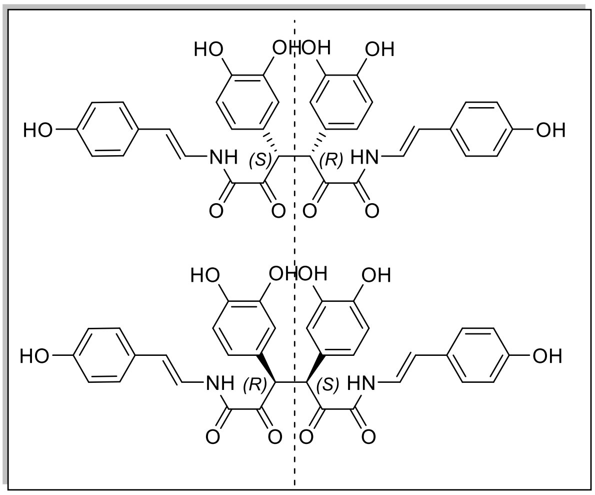

A meso compound (3*R*,4*S* or 3*S*,4*R*) of **3** would be achiral

Figure S41. Relative configuration of compound **3**

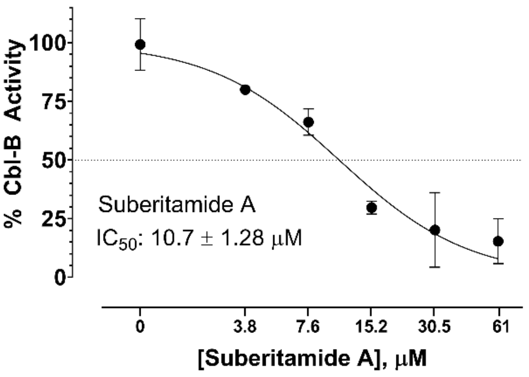

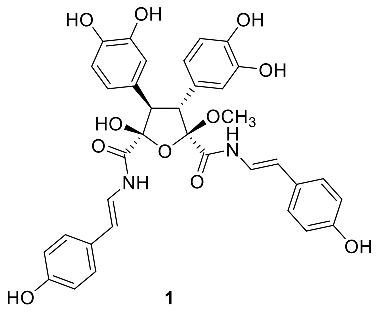

1

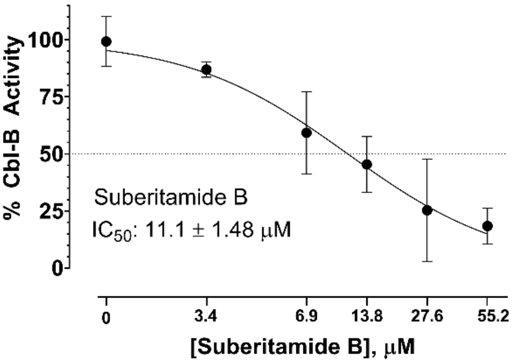

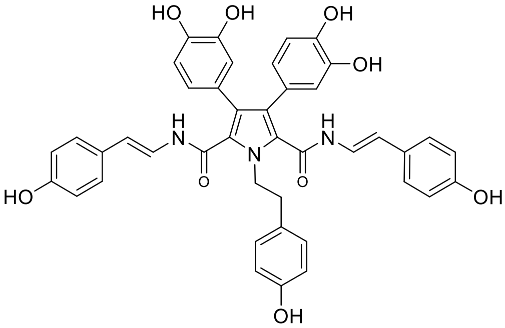

2

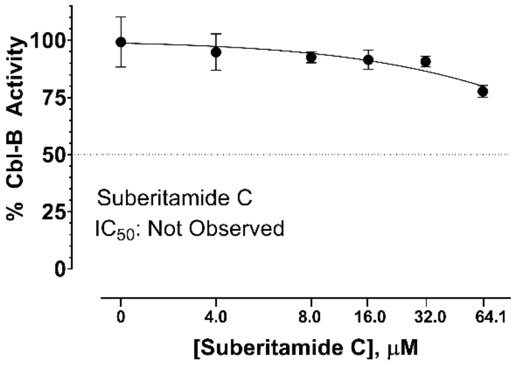

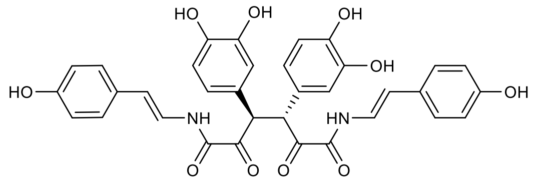

3

Figure S42. Dose-response curves of Cbl-b inhibitory activity for **1-3**
